# Small Multitarget Molecules Incorporating the Enone Moiety

**DOI:** 10.3390/molecules24010199

**Published:** 2019-01-07

**Authors:** Thalia Liargkova, Nikolaos Eleftheriadis, Frank Dekker, Efstathia Voulgari, Constantinos Avgoustakis, Marina Sagnou, Barbara Mavroidi, Maria Pelecanou, Dimitra Hadjipavlou-Litina

**Affiliations:** 1 Department of Pharmaceutical Chemistry, School of Pharmacy, Faculty of Health Sciences, Aristotle University of Thessaloniki, Thessaloniki 54124, Greece; thalialiargkova@yahoo.gr; 2 Department of Pharmaceutical Gene Modulation, Groningen Research Institute of Pharmacy, University of Groningen, Antonius Deusinglaan 1, 9713 AV Groningen, The Netherlands; nikolaoselef@hotmail.com (N.E.); f.j.dekker@rug.nl (F.D.); 3 Department of Pharmaceutical Technology and Pharmaceutical Analysis, School of Pharmacy, University of Patras, Rio Patras 26504, Greece; efiv48@hotmail.com (E.V.); avgoust@upatras.gr (C.A.); 4 Institute of Biosciences and Applications, National Center for Scientific Research “Demokritos”, Agia Paraskevi, Athens 15310, Greece; sagnou@bio.demokritos.gr (M.S.); bmavroidi@bio.demokritos.gr (B.M.); pelmar@bio.demokritos.gr (M.P.)

**Keywords:** chalcones, *bis*-ethers, *bis*-chalcones, multitarget, lipoxygenase inhibitors, acetylcholinesterase inhibitors, β-amyloid peptide, Alzheimer

## Abstract

Chalcones represent a class of small drug/druglike molecules with different and multitarget biological activities. Small multi-target drugs have attracted considerable interest in the last decade due their advantages in the treatment of complex and multifactorial diseases, since “one drug-one target” therapies have failed in many cases to demonstrate clinical efficacy. In this context, we designed and synthesized potential new small multi-target agents with lipoxygenase (LOX), acetyl cholinesterase (AChE) and lipid peroxidation inhibitory activities, as well as antioxidant activity based on 2-/4- hydroxy-chalcones and the *bis*-etherified *bis*-chalcone skeleton. Furthermore, the synthesized molecules were evaluated for their cytotoxicity. Simple chalcone **b4** presents significant inhibitory activity against the 15-human LOX with an IC_50_ value 9.5 µM, interesting anti-AChE activity, and anti-lipid peroxidation behavior. *Bis*-etherified chalcone **c12** is the most potent inhibitor of AChE within the *bis*-etherified *bis*-chalcones followed by **c11**. *Bis*-chalcones **c11** and **c12** were found to combine anti-LOX, anti-AchE, and anti-lipid peroxidation activities. It seems that the anti-lipid peroxidation activity supports the anti-LOX activity for the significantly active *bis*-chalcones. Our circular dichroism (CD) study identified two structures capable of interfering with the aggregation process of Aβ. Compounds **c2** and **c4** display additional protective actions against Alzheimer’s disease (AD) and add to the pleiotropic profile of the chalcone derivatives. Predicted results indicate that the majority of the compounds with the exception of **c11** (144 Å) can cross the Blood Brain Barrier (BBB) and act in CNS. The results led us to propose new leads and to conclude that the presence of a double enone group supports better biological activities.

## 1. Introduction

“Small-molecule drug” refers to a compound with a low molecular weight, able to enter cells easily, affecting other biological molecules, such as proteins, enzymes, etc., and may cause the death of cancer cells. They strongly differ from drugs that have large molecular weight, such as monoclonal antibodies, which are not able to get inside cells very easily. Chalcones belong to the group of small molecules, known as α, β-unsaturated ketones, displaying a very large number of biological activities [1]. Numerous published reviews underline the medicinal significance of the enone moiety of chalcones [2,3,4,5,6]. This conjugation of the double bond with the carbonyl moiety seems to be responsible for the biological activities of chalcones, as removal of the enone group renders them inactive. Chalcones represent a key structural scaffold for many synthetic and natural products. While a number of synthetic processes have been reported for the synthesis of chalcones, the general and more widely used synthetic route involves Claisen–Schmidt condensation under acidic or basic homogeneous conditions [7,8,9,10,11]. Changes in their structure have offered a high degree of diversity which has been extremely useful and highly advantageous for medicinal chemistry purposes aiming to develop new therapeutic agents with improved pharmacokinetic properties, as well as a better therapeutic profile.

The formation of reactive oxygen species (ROS) is characteristic of aerobic organisms. However, in many pathophysiological conditions the excessive production of ROS overwhelms the natural antioxidant defense mechanisms leading to an imbalance widely known as oxidative stress (OS). Antioxidants have been found to prevent and offer therapeutic benefits on inflammation-generated oxidative stress which is closely related to the lesions of Alzheimer’s disease (AD) [12]. Nowadays the involvement of free radicals in the AD brain pathology includes the presence of elevated levels of protein oxidation, lipid peroxidation products and oxidative mitochondrial damage [12]. Hydroxy-chalcones have exhibited a very strong antioxidant profile which has been directly related to the presence of the α, β**-double bond and the hydroxyl moiety [2,3,4,5,6]. Therefore, they may be considered as promising structural templates to design potential therapeutic agents against the ROS-related AD pathology.

Acetyl cholinesterase (AChE) is a serine hydrolase mainly found at neuromuscular junctions and cholinergic brain synapses with the biological role to terminate impulse transmission at cholinergic synapses by rapid hydrolysis of the neurotransmitter. The inhibition of brain AChE presents a very attractive and highly promising therapeutic target in AD treatment strategies.

Chronic inflammation is a common phenomenon present in the background of multiple neurodegenerative diseases, including AD. Previous studies showed that the key regulatory enzymes in the eicosanoid pathway, i.e., cyclooxygenase-2 and 5-, 12-, and 15-lipoxygenases, appear to have an important role in mediating the pro-inflammatory responses, providing further evidence that, in the future, it may be possible to manipulate pro-inflammatory pathways for therapeutic purposes [13]. Lipoxygenases (LOXs) are dioxygenases which catalyze the stereoselective addition of oxygen to arachidonic acid (AA). They are characterized by the presence of a non-heme iron in their structure. LOXs peroxidize membrane lipids causing structural changes to the cell [14]. The most abundant LOX isoforms in the central nervous system are 12/15-LOX. The metabolites of these enzymes, 12(S)-HETE and 15(S)-HETE, are important secondary messengers in synaptic transmission and are involved in learning and memory processes. 12/15-LOX have been described abundantly in neurons and in some glial cells throughout the cerebrum, hippocampus, and basal ganglia [15,16]. Oxidative stress and inflammatory reactions have been related with the both up-regulation of 12/15-LOX expression levels and activity [15,17]. The mammalian reticulocyte 15-LOX-1 is the major enzyme which is responsible for membrane lipid peroxidation [18]. Experiments on animal AD models have provided evidence on the importance of 15-LOX and have demonstrated its role in the etiopathology of AD. Furthermore, 15-LOX has been found to promote brain cell survival through the synthesis of neuroprotectin D1 [19,20,21], an antiapoptotic and neuroprotective docosahexaenoic acid.

Moreover, the 5-LOX inhibitor, Zileuton, reduced Aβ formation in embryonic fibroblasts. [22] Wang et al. [23] showed that 12-LOX activation plays a key role in oxidative injury due to glutathione (GSH) depletion. Inhibitors for 5-lipoxygenase (5-LOX) and cyclo-oxygenase 2 (COX-2 and 5-, 12-, and 15-LOX-inhibitor in astrocytes reduced significantly IL-6 secretion, compared to exposed glial cells without inhibitors [13]. Treatment with an inhibitor of all LOXs, namely nordihydroguaiaretic acid, or 5-LOX inhibitors, such as zileuton, BWB70C, and inhibitors of 12/15-LOX, baicalein and AA-861 (also an inhibitor of 5-LOX) significantly reduced, in a concentration-dependent manner, the level of lipid and protein oxidation [24].

For many years, researchers have been struggling to develop highly specific compounds against a particular target. However, the “one drug-one target” therapies failed to demonstrate clinical efficacy in multifactorial disorders. Consequently, the treatment of these multi-factorial diseases, such as AD, may have to depend on and be the result of the concurrent interference with more than one pathological pathway, to exert therapeutic benefits. In the last decade the idea of a drug to be “promiscuous” has been correlated with its ability to selectively target multiple cellular processes and to treat diseases that stem from a combination of biological parameters. Following this approach, the researchers succeeded in having one single molecule hit multiple targets that participate in the pathogenesis of a multifactorial disease [25]. In this context, a single multi-target drug may have distinctive advantages over drug combination therapies [26] and as a result an increasing number of therapeutic strategies that are based on poly-pharmacology have been proposed for AD [27,28]. Therefore, it is evident that the treatment of AD could benefit from the use of multipotent drugs that present free radical scavenging, anti-lipid peroxidation, anti-inflammatory, and AChE inhibitory activity.

**Figure 1:**
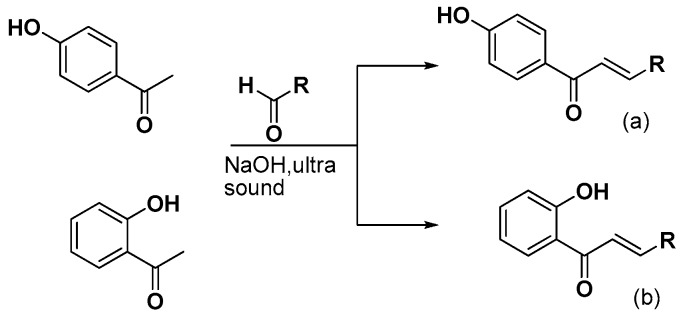
Synthesis of 2-hydroxy- and 4-hydroxy-substituted chalcones.

**Figure 2:**
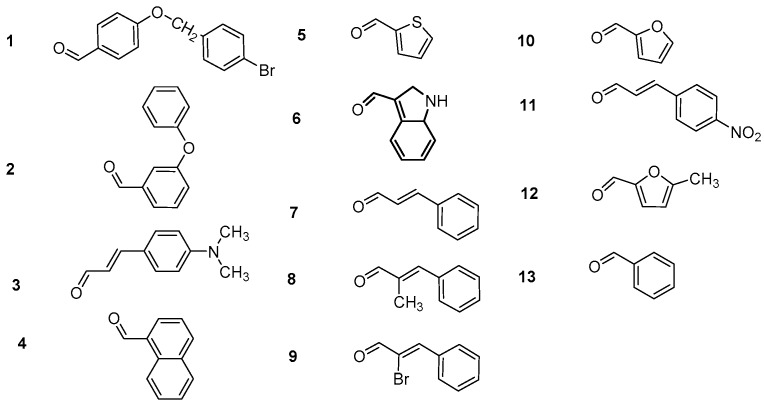
Structures of the used aromatic aldehydes.

**Figure 3:**
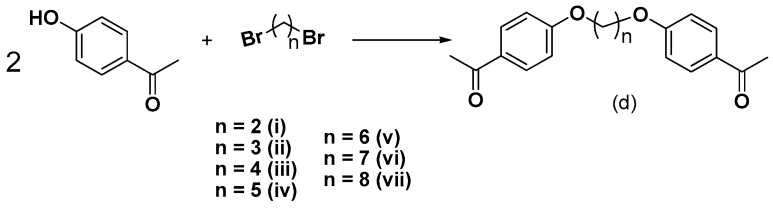
Synthesis of the *bis*-ethers.

**Figure 4:**
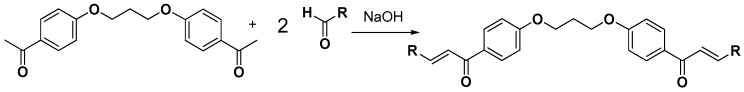
Synthesis and general structure of *bis*-etherified *bis*-chalcones.

**Figure 5:**
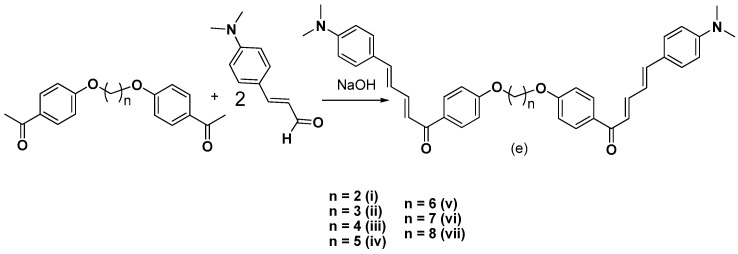
Synthesis of the dimethyl amino substituted *bis*- etherified *bis*-chalcones.

Herein, we continue our efforts [29] to design and synthesize small biologically active molecules and for this scope, the chalcone structural template was exploited as a privileged scaffold for the design of multi-target agents. The synthesis of simple chalcones **a1**–**4**, of the *bis*-etherified chalcones **c1**–**4** and of the intermediate ether **dii**, as well as their antioxidant activities (DPPH, AAPH, ABTS^•+^), cytotoxicity, and inhibition against lipoxygenase and acetylcholinesterase have been already published [29]. Several substituted aromatic and heteroaromatic aldehydes have been used in order to evaluate the effect of steric and electronic parameters on the biological activity and to optimize the activities through systematic modification of the substituents. Thus, we present the synthesis and biological evaluation of a series of 2-hydroxy- and 4-hydroxy-chalcones (Figure 1 and Figure 2), diversely substituted, as well as of a series of *bis*-chalcones combined together through an **−O(CH_2_)_n_O−** linkage leading to the structures of *bis*-ethers (Figure 3, Figure 4 and Figure 5) in which the effect of the length of the chain was studied.

## 2. Results and Discussion

### 2.1. Chemistry

The 4-hydroxy-substituted chalcones **a**(**1**–**13**) and the 2-hydroxy-substituted chalcones **b**(**1**–**4**, **7**–**9**, **11**, and **13**) were successfully synthesized via Claisen-Schmidt condensation, using 20% KOH as a catalyst in a US-bath. Figure 1 shows a generalized synthetic route for the preparation of chalcones of **a** and **b** series, whereas the structures of the various substituted aromatic aldehydes used for those derivatizations are presented in Figure 2. During the course of these reactions, the use of the US-bath was found to significantly affect the reactivity of the starting material, as well as the yields of chalcone formation. For the synthesis of *bis*-etherified double chalcones **c** and **e**, 4-hydroxyacetophenone was successfully dimerized to form *bis*-ethers **d**(**i**–**vii**), by means of a Williamson reaction in dry acetone and in the presence of the desired 1,ω-dibromoalkane, under basic conditions and reflux, as shown in Figure 3. Subsequent reaction of **dii**, for which a 3C-ether link was employed between the two 4-hydroxyacetophenone units, with the aromatic aldehydes of Figure 2 under Claisen-Schmidt conditions afforded chalcones **c**(**1**–**13**) is shown in Figure 4. Finally, prompted by the encouraging results of our previous work on *bis*-etherified chalcone **c3** [29], which resulted from the reaction of the 4-dimethylamino(phenyl)acrylaldehyde with **dii**, and exhibited a very potent anti-lipid peroxidation activity (100%), a series of the corresponding *bis*-etherified double chalcones **e**(**i**–**vii**) were synthesized in a similar manner, as shown in Figure 5. These molecules were anticipated to reveal the effect of the carbon chain length of the *bis*-ether between the two 4-dimethylamino(phenyl)acryl-derived chalcone units.

Their structures are confirmed spectroscopically (IR, ^1^H-NMR, ^13^C-NMR, and LC-MS) and by elemental analysis. All the simple chalcones of series **a** and **b** present the characteristic absorption in the IR (KBR cm^−1^ 3280–3550 (O–H), 1720 (C=O), 1625 (C=C)). ^1^H-NMR spectroscopy revealed through integration the right analogy of aromatic and C*H*= protons. The results are consistent with the proposed (*Ε*) structures and are in agreement with our previous findings [29]. All the derivatives were taken in satisfactory yields (over 70%). The physicochemical properties of the products are described in the experimental session.

We did not succeed to synthesize *bis*-etherified *bis*-chalcones from the 2-hydroxy-substituted chalcones. The chromatographic data and the spectrometric studies verified the failure of the reactions due to stereochemical reasons.

Several 2-hydroxy- and 4-hydroxy-chalcones and derivatives are known as anti-tumor anti-inflammatory, anti-parasites, anti-depressive, anticonvulsant, antimicrobial, antinociceptives, and nitric oxide synthase inhibitors, associated with diseases such as Alzheimer’s and Huntington’s [30,31]. Biological results from 3-hydroxy chalcones and derivatives have also been reported. The results from simple amino ethers derived from 3-hydroxy chalcones suggested that amino alkyl side chain of chalcone dramatically influenced the inhibitory activity against AChE. Among them, the structural combination of the 4,6-diamino-1,2-dihydro-1,3,5-triazine and chalcone scaffolds via flexible diether linkers of varying lengths were successfully synthesized and characterized [32]. The resulting compounds were evaluated as dual-target inhibitors of dihydrofolate reductase (DHFR) and thioredoxin reductase (TrxR), revealing the influence of linker length on biological activities.

It is worth mentioning, and aiming to rationalize our choice of aldehydes used in this study (Figure 2) that derivatives of some heterocyclic chalcones bearing thiofuran, furan, and quinoline moieties have presented interesting biological activities [33]. Hence, aldehydes **4**, **5**, **10**, and **12** were used, whereas 3-hydroxychalcones and their substituted benzo-ethers have been tested as multifunctional non-purine xanthine oxidase inhibitors [34] prompting us to use aldehydes **1**, **2**, and **13**. Diarylpentanoid analogues evaluated as nitric oxide inhibitors [35] were found to inhibit superoxide anion and elastase release in human neutrophils [36], have inspired us to employ aldehydes **3**, **7**–**9**, and **11** for the desired chalcone formation.

### 2.2. Physicochemical Studies

#### 2.2.1. Experimental Determination of Lipophilicity as R_M_ Values

It is well-documented and discussed in the literature that lipophilicity is a very significant physicochemical property which may critically affect distribution, bioavailability, metabolic activity and elimination, all the useful characteristics for the kinetics of biologically active compounds. Thus, we attempted to determine experimentally the lipophilicity of the synthesized compounds from the RPTLC method [37] as R_M_ values. The method is considered to be a reliable, fast and convenient method for expressing lipophilicity. Moreover, many calculated methods were used in order to predict the logP value [38,39,40].

#### 2.2.2. In Silico Determination of Lipophilicity Values as cLogP

The CLOGP program of BioByte Corp. [41] (BioByte Corp., Claremont, CA, USA) was used for the in silico calculation of lipophilicity values in n-octanol buffer. Lipophilicity was theoretically calculated as cLogP values.

The attempt to correlate together in one equation simultaneously the theoretically calculated lipophilicity values calculated with C-QSAR (as cLogP*)* and the R_M_ values of all the compounds did not succeed. This disagreement may have been the result of various factors, such as the specific chromatographic behavior of the compounds (e.g., different solvation, silanophilic interaction, H-bridges, etc.), causing such limitations. Thus, the different nature of the hydrophilic and lipophilic phases used in the two systems (*n*-octanol: water for the theoretically calculated values and methanol-water: nujol for the RPTLC experiment) must be considered. It is difficult to obtain reliable experimental logP values outside the range of about 5 < logP < 7; therefore, it is likely that a calculated logP value using a model is more accurate than an experimental [42].

However, we succeeded to correlate the calculated lipophilicity values (as cLogP*)* and the experimental R_M_ values for the simple chalcones in the following statistically significant equation:

R_M_ = 0.431 (±0.198) cLogP − 2.108 (±0.753) N = 11, r = 0.854, q^2^ = 0.592, s = 0.257, F_1, 9_ = 24.266, α = 0.01 (1)

#### 2.2.3. Molecular Properties Prediction—Lipinski “Rule of Five”

Computational methods have emerged as a powerful strategy for the prediction of human pharmacokinetic properties. In this regard, a variety of useful in silico models have been developed with different levels of complexity for the screening of data sets of compounds, creating tools that are faster, simpler, and more cost-effective than traditional experimental procedures [43].

Thus, chemical structures and SMILES notations of the title compounds were obtained and entered in the online Molinspiration software version 2016.10 (www.molinspiration.com, Bratislava, Slovak Republic, Bratislava University) [44] to calculate various molecular properties e.g., partition coefficient (log P), topological polar surface area (TPSA), hydrogen bond donors and acceptors, rotatable bonds, number of atoms, molecular weight, and violations of Lipinski’s rule of five, in order to evaluate the drug likeness of chalcones and intermediates [45]. In the discovery, “the rule of five” predicts that poor absorption or permeation is more likely when there are more than five H-bond donors, 10 H-bond acceptors, the molecular weight (MW) is greater than 500, and the calculated Log P is greater than 5. It is a rule of thumb to evaluate drug-likeness or determine if a chemical compound with a certain pharmacological or biological activity has properties that would make it a likely orally active drug in humans.

LogP values of all the title compounds except **a3**–**13, b3**–**13** and **di**–**div**, were found to be more than 5 and are in clear violation of Lipinski’s rule of five, suggesting poor permeability across cell membrane. As shown in Table 1, logP values range from 2.41 to 9.44. LogBB (BB, Blood Brain Barrier) is another important in silico parameter to identify CNS active agents. For the calculation of the logBB values we use the cLogP values. For in silico prediction [46], compound with logBB value more than 0.3 is considered to have high absorption through BBB whereas between 0.3–0.1 and less than −0.1 is considered to be moderate and less absorbed through BBB. LogP values of tacrine and nordihydroguaiaretic acid (NDGA) the standard drugs were found to be well under 5 justifying their oral use. Twenty-nine (29) derivatives were found to present molecular weight less than 500. Thus, these molecules could be easily transported, diffused, and absorbed in comparison to large molecules. Counting the number of hydrogen bond acceptors (O and N atoms) and the number of hydrogen bond donors (NH and OH) in the synthesized compounds it seems that both follow the Lipinski’s rule of five (less than 10 and 5, respectively). Within the series of the examined derivatives, only compounds **a3**–**13** and **b3**–**div** seem to be orally active in accordance to Lipinski’s rule of five.

Topological polar surface area is highly correlated with the hydrogen bonding of a compound. It is used as a significant indicator of the bioavailability of a bioactive molecule. TPSA of the derivatives was observed in the range of 37.3–144.26 Å and is well below the limit of 160 Å indicating good oral bioavailability. The upper limit for TPSA for a molecule to penetrate the brain is around 90 Å. Results indicate that all structures with the exception of **c11** (144 Å) can cross the BBB.

### 2.3. Biological Assays

**Figure 6:**
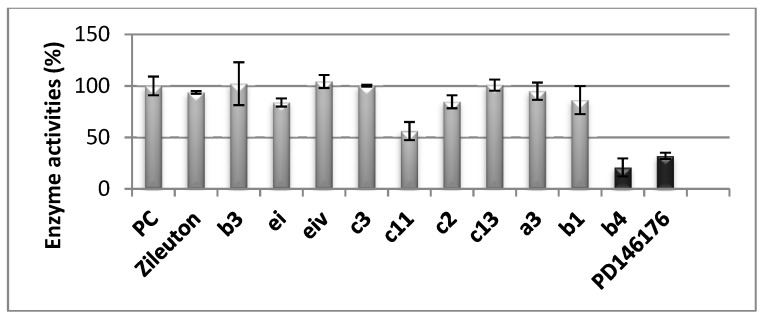
The residual enzyme activities % (human h-15-LOX-1) resulting from the tested compounds at 50 µM.

**Figure 7:**
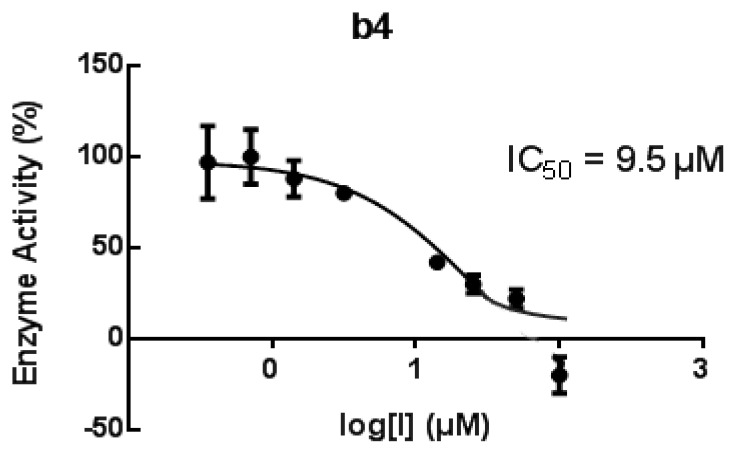
IC_50_ value for human h-15-LOX-1 from compound **b4.**

A number of chalcone derivatives have been obtained and evaluated herein as pleitropic potential antioxidant agents, inhibitors of AChE and Lipoxygenase in vitro. Among them we included also **a1**–**4**, **c1**–**4** and **dii** for which earlier we performed a number of in vitro assays [29]. For the sake of comparison, we include them in the tables and in the discussion, and simultaneously we investigate their behavior in some additional assays. Numerous studies in patients with Alzheimer’s disease (AD) showed a significant enhancement of lipid peroxidation in their brain. Thus, radical scavengers and antioxidants could offer in the treatment of AD patients targeting brain lipid peroxidation. As we already mentioned free radicals are highly implicated in lipoxygenase induction, inflammation, and neurodegenerative diseases such as AD and cancer. Compounds possessing these activities in combination with antioxidant activity might be protective against these diseases and lead to active and useful agents. Thus, it would be interesting to determine their antioxidant activities in comparison to well-known antioxidants, i.e., nordihydroguaiaretic acid (NDGA) and Trolox (Table 2 and Table 3, Figure 6 and Figure 7).

In vitro antioxidant activity has been determined with the aid of a large number of different assays in order to take into consideration significant factors such as solubility or steric hindrance which may be important in different milieu, and reveal their significance. All the recorded methods are associated with the generation of various radicals. Two approaches are referred: (i) the scavenging by hydrogen- or electron donation of a preformed free radical, and (ii) the presence of an antioxidant system during the generation of the radical.

The chalcones were studied for the antioxidant activity (reducing activity RA%) by the use of the stable free radical 2,2-diphenyl-1-picrylhydrazyl radical (DPPH) at concentrations 0.05 mM after 20 and 60 min (Table 2). In this procedure, the dominant chemical reaction involved is the reduction of the DPPH radical by single electron transfer from the antioxidant. Thus, phenolic compounds, e.g., nordihydroguaiaretic acid (NDGA), giving phenoxide anions are effective antioxidants. Considering the simple chalcones of **a** series, the 4-OH substituted derivatives, exhibited very low/limited reducing activity. From the derivatives of **b** series, the 2-OH substituted chalcones, only compound **b3** (72%) the 4-dimethyl-amino-phenyl acryl derivative presented very high ability, followed by **b4**, whereas all the other simple tested chalcones exhibited low or no activity. For the sake of comparison, we measured the RA% values for the intermediate double ethers **d** which were included in the Table 2. They did not present any activity by themselves.

The decolorization assay is used to evaluate the antioxidant activity. The ABTS^•+^ cationic radical is derived from the direct oxidation of ABTS by potassium persulfate. No involvement of an intermediary radical is observed. The addition of electron-donating antioxidants reduce ABTS^•+^. The radical is formed prior to the addition of the antioxidant and does not take place continually in the presence of the antioxidant. Again, we observed very low or no antioxidant activities for both series **a** and **b**. Chalcone **b3** showed the highest activity among the **a** and **b** simple chalcones. The intermediate ethers **d** did not show any activity. All the *bis-*etherified *bis-*chalcones presented limited activity with the exception of compounds **c3**, **ei**, **eiv**, **evi**, and **evii** (Table 2). The structural characteristics highlight the importance of the 4-dimethyl-amino-phenyl) acryl group (Figure 2, structure 3). The presence of this moiety is correlated with higher antioxidant activity. The role of the length of the ether chain within the derivatives of subgroup **e** is not well defined, e.g., **c3** (99%, n = 3), **ei** (98%, n = 2), **eiv** (73%, n = 5), **ev** (25%, n = 6), **evi** (79%, n = 7), and **evii** (88%, n = 8).

The water soluble 2,2′-azobis(2-amidinopropane) hydrochloride (AAPH) generates in vitro free radicals through spontaneous thermal decomposition. The experimental conditions employed in or study significantly resemble to cellular lipid peroxidation due to the activity of the peroxyl radicals. Overall, chalcones **a**, **b**, and **c** present anti-lipid peroxidation activity. Chalcones **a5**–**13** exhibit moderate activity at 100 µM concentration. The **b1**, **b3**, and **b4** (82–100%) are the most active inhibitors among the **b** chalcones. The inhibiting activities for the rest **b** chalcones ranged from 15–44%. The intermediate ethers are moderate inhibitors. It seems that the *bis-*chalcones **c** are more potent anti-lipid peroxidation agents compared to the corresponding simple chalcones. This might be correlated with the presence of the double **C=C–COCH_3_** group. Among the **e** *bis-*etherified *bis-*chalcones, **evii** exhibited the lowest activity followed by **ev**. Both have a long ether chain in their structure.

We used liposomes to investigate the antioxidant activity of the *bis-*chalcones. The undertaken results showed high potency for **c3**, **c12**, **eiv**, and **evi**. The observed % inhibition activities were found to be in agreement with the results taken from the AAPH assays. Considering chalcones **c2**, **c4**, and **ei**, the results obtained were not able to provide any additional information.

Lipoxygenases (LOXs) catalyze the metabolism of arachidonic acid to leukotrienes, important inflammatory mediators [47]. Recently published research showed that AD brains had higher 5-LOX protein levels than did healthy controls [48]. It has been found the activation of brain lipoxygenases is an early event in the pathogenesis of Alzheimer’s disease [49]. Furthermore 12/15-LOX activity was increased in pathologically affected areas of AD brains [50] and, consequently, 5-lipoxygenase (5-LOX) acts as a modulator of Aβ peptides formation in vivo [48]. Some new studies points to a new role for 5-LOX in regulating endogenous tau metabolism in the central nervous system supporting the hypothesis that the inhibition of 5-LOX could be beneficial for Alzheimer’s disease-related tau neuropathology [51].

In our experiments we used soybean lipoxygenase. Designing agents to modulate activities of the variety of so closely homologous enzymes, such as different LOXs, require an intimate knowledge of their 3D structures, as well as information about metabolism of the potential xenobiotics. Thus far only the structures of soybean isozymes LOX-1 and LOX-3 have been determined for native enzymes, and several structures of their and rabbit 15-LOX (from reticulocytes) molecular complexes with inhibitors are known. Due to lack of sufficiently-purified human enzymes most of the research has been done on soybean LOX [52,53]. Although this enzyme is not identical to the mammalian one, it seems that there is a sufficient qualitative correlation between values for enzyme inhibition with the two enzymes [54,55,56].

Thus, we used the isozyme LOX-1 in our assays which only uses free fatty acids as substrates [57] exhibits maximal activity at pH 9.0 and converts linoleic acid into to 13-hydroperoxylinoleic acid [57] producing a conjugated diene that absorbs at 234 nm.

LOXs contain a “non-heme” iron per molecule in the enzyme active site as high-spin Fe^2+^ in the native state and the high spin Fe^3+^ in the activated state. Nordihydroguaiaretic acid (NDGA), referred as known inhibitor of soybean LOX, has been used as a reference compound (IC_50_ 0.5 µM/93% at 100 μM).

Perusal of the %/IC_50′s_ inhibition values (Table 3) shows that the simple 2-OH substituted chalcones are less potent than the corresponding 4-OH-substituted. Chalcone **c11** was found to be the most active inhibitor within all the series followed by: **c11** > **c6** > **b1**, **b3** > **ei > c13**, **b4** > **evi** > **c10** and the inhibitory activities in terms of IC_50_ values were in the range 50–100 µM. Compounds **b1**, **b3** and **c13**, **b4** were found to be almost equipotent inhibitors The chain length of the ether linker does not affect the degree of inhibition of the soybean LOX within the **e** compounds and that the three-carbon chain provides a structural advantage over the longer (seven-carbon) or shorter (two-carbon) linkers. For the sake of comparison, the intermediate *bis-*ethers **d** were tested and found to be inactive.

Lipophilicity is referred to as an important physichochemical property for lipoxygenase inhibition [58]. However, in this dataset lipophilicity does not seem to correlate with LOX inhibition, since the most active inhibitors present high lipophilicity value, but the activity does not exhibit any apparent trend related to lipophilicity values. Thus, the presence of the two enone groups in combination to the ether linkage is correlated with higher inhibitory activity. The length of the ether chain seems to be of minor importance. The results derived from the chalcones, for which aldehydes **1**–**4** [29] and **11** and **13** were used as synthetic blocks, were more significant. The role of any other specific structural characteristics are not well defined.

A large number of bioactive compounds inhibit lipoxygenase activity and, as a consequence, several mechanisms of action have been proposed [59]. However, most of the LOX inhibitors are antioxidants or free radical scavengers. Thus, LOX inhibitors: a) can reduce the iron species in the active site to the catalytically inactive ferrous form or b) can act as ligands for Fe^3+^.

Herein, the results from the anti-lipid peroxidation activity studies support the anti-LOX activity for the significantly active *bis-*chalcones **c11**, **c6**, **b1**, **b3**, **ei**, **c13**, **b4**, **evi**, and **c10**. From the literature it is clear that there are no strict structural requirements for lipoxygenase inhibition, thus. There is no universally accepted approach to evaluate the relative potency of different substances to cause lipoxygenase inhibition.

Due to the key role of 15-LOX-1 in AD, we decided to test **b3**, **ei**, **eiv**, **c3**, **c2**, **c13**, **a3**, **b1**, and **b4** as inhibitors of recombinant h-15-LOX-1, since these chalcones exhibited significant results against soybean LOX (Figure 6). PD-146176 has been reported by Parke-Davis (now Pfizer) [60] as an inhibitor of h-15-LOX-1. The residual enzyme activity was measured after 10 min pre-incubation with the tested compounds at room temperature. As reference standard compounds the known inhibitors, PD-146176 (15-LOX) and Zileuton (5-LOX), were also tested. The results of the screening are shown in Figure 6. It seems that the simple chalcone **b4** presents the highest activity within the group and in comparison to the reference **PD-146176.** Compound **c11** follows**.** Thus, we found it interesting to determine its IC_50_ value, which was found to be 9.5 µM, whereas for **PD-146176** it was 16 µM.

We investigated, in vitro, the inhibitory activity of **a**, **b**, **c**, and **e** derivatives on acetylcholinesterase activity using acetylthiocholine as a substrate [29]. The potential of these compounds to act as acetylcholinesterase inhibitors can be considered beneficial for their prospective nootropic action and may contribute to the mechanisms of action of reported structurally related hydroxyl chalcones [61].

All the **c** and **e** *bis-*chalcone derivatives present significant IC_50_ values as it is shown in Table 3 with the exception of **c6**, **eiii**, and **ev**. The simple **a** chalcones are less active with a % inhibition range 7–48% (Table 3). It should to be noticed that simple chalcones **b3** and **b4** presented high inhibitory activities. They exhibited 100% or 82% inhibition at 0.001 µM, whereas **b1** gave IC_50_ value 100 µM. Unfortunatelly, we did not succeed to determine the IC_50_ values of **b3** and **b4**, which constantly presented high inhibition independently of the decrease of the concentration. All the rest of the **b** chalcones were much less efficient in their inhibitory action. The *bis-*chalcone **c6** did not present any activity. On the contrary, *bis-*chalcone **c12** was found to be the most active, followed by **c12** > **c7** > **ei** > **c8** > **evi** > **eiv** > **c10** > **c5**.

Again it is observed, as it has been found from our published results [29], that the transformation of a simple to a *bis-*chalcone with an ether linkage leads to more potent analogues (**a3**/**c3**) [29]. The chain length of the ether linker does not affect the inhibition of AChE induced by **e** compounds and that the three-carbon chain provides structural advantage over the longer (seven-carbon) or shorter (two-carbon) linkers.

Perusal of logP and IC_50_ or % AChE values in Table 3 reveal that the role of lipophilicity on the inhibition of AChE is also not well defined in this series of compounds.

The cytotoxicity of the synthesized derivatives against L929 mouse fibroblasts cells was determined using the propidium iodide (PI) fluorescence method [29] in the presence of different concentrations (1–100 μM) of these compounds. The results are presented in Table 4 in the form of the % cell survival values as PI% for the examined compounds. All tested chalcones **a**(**5**–**12**) showed low cytotoxicities in the whole area of concentrations examined (from 1 μM to 100 μM), with the noticeable exception of **a7**, **a11**, and **a12**. Considering the *bis-*chalcone ethers **c5**, **c6**, **c9**, and **c10**, they showed low cytotoxicity. Chalcones **c7**, **c8**, **c11**, and **c12** follow. For these compounds cell toxicity started increasing significantly at the concentration of 20 μM, reaching the highest values at 100 μM.

**Figure 8:**
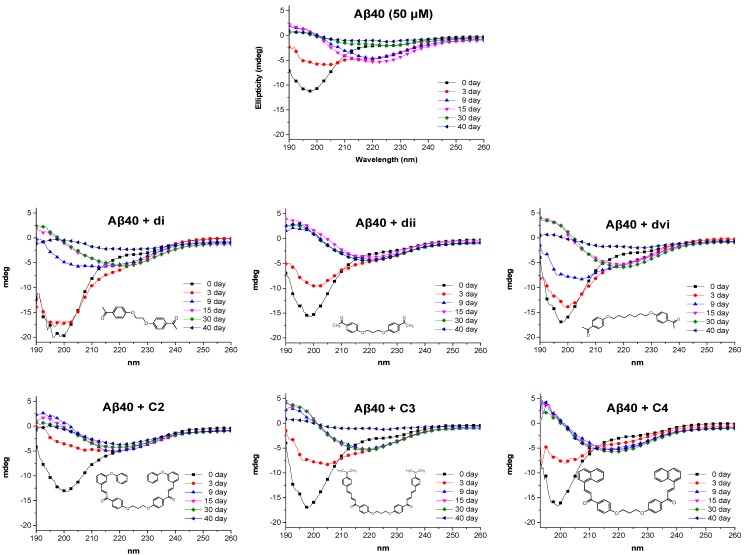
CD spectra of Aβ40 (50 µM) in phosphate buffer (PB 10 mM, pH 7.33) in the absence or presence of 50 µM of intermediate *bis*-ethers **di**, **dii**, **dvi**, and *bis*-etherified *bis*-halcones **c2**, **c3**, and **c4** (1:1 ratio). Spectra were recorded for a period of 40 days at 33 °C. Representative spectra from n= 3 independent experiments are presented.

The pathogenesis of Alzheimer’s disease (AD) is widely associated with the aggregation of β-amyloid peptide (Aβ) either to soluble oligomers or to higher-order polymeric insoluble fibrils [62,63]. As a result, one of the main therapeutic strategies against AD targets the aggregation process of Aβ, and a great number of small molecules are being investigated for their potential to intervene in the assembly of Aβ and inhibit its neurotoxicity in vitro and in vivo [64]. Circular dichroism (CD) spectroscopy is commonly used to probe the secondary structure of polypeptides and to monitor conformational changes and interactions of polypeptides with small molecules. The typical aggregation process of Aβ produces characteristic CD spectra over time that reflect the conformational changes that occur as the random coil monomers are converted into β-sheet oligomers, and then gradually to larger β-sheet formations that finally produce amyloid fibrils [65]. CD is, therefore, a valuable tool to monitor the aggregation process of Aβ and to study the effect of potential aggregation inhibitors. To further investigate the pleiotropic effects of chalcones against AD, selected compounds from our library, were subjected to CD studies in order to evaluate their potential to interfere with the aggregation process of Aβ40. Figure 8 shows the results of the CD experiments performed over a period of 40 days. The aggregation spectra of plain Aβ40 show at day 0 the typical random coil peak (negative maximum at 198 nm) the intensity of which gradually decreases as the peptide forms β–sheet structures (negative maximum at 222 nm, day 15) that further aggregate to higher order polymeric fibrils. The precipitation of the insoluble fibrils from solution eventually results in the loss of CD signal (days 30 and 40) [66]. As can be seen in Figure 8, the presence in the Aβ40 solution of intermediate *bis-*ethers **di** and **dvi,** apart from a noted delay in the overall process, did not significantly change the fibrillization course of Aβ40, and the CD signal almost reached baseline at day 40. On the contrary, addition of *bis-*ether **dii** modified the aggregation course of Aβ40 by stabilizing a β-sheet structure that did not seem to evolve further. This suggests that the chain length of the ether linker affects the degree of interaction of the compounds with Aβ and that the three-carbon chain provides a structural advantage over the longer (seven-carbon) or shorter (two-carbon) linkers. In view of the above results, chalcone derivatives bearing a three-carbon chain linker, specifically **c2**, **c3**, and **c4**, were subsequently studied in order to assess the effect of further derivatization and the presence of the chalcone moiety in the structure. As shown in Figure 8, in the case of **c3**, no significant effect on the aggregation process of Aβ40 was observed, while on the contrary the presence of compounds **c2** and c**4,** which include the intact chalcone moiety in their structure, stabilized β-sheet assemblies that did not evolve further into insoluble fibrillar aggregates, as was also observed in the case of compound **dii**. Overall, our CD study identified three new structures capable of interfering with the aggregation process of Aβ, out of which compounds **c2** and **c4** display additional protective actions against AD and add to the pleiotropic profile of the chalcone derivatives.

## 3. Experimental Section

### 3.1. Materials and Instruments

All chemicals, solvents, and chemical and biochemical reagents were of analytical grade and purchased from commercial sources (Merck, Merck KGaA, Darmstadt, Germany, Fluka Sigma-Aldrich Laborchemikalien GmbH, Hannover, Germany, Alfa Aesar, Karlsruhe, Germany and Sigma, St. Louis, MO, USA). Soybean lipoxygenase, sodium linoleate, and 2,2-azo*bis*-(2-amidinopropane) dihydrochloride (AAPH) were obtained from Sigma Chemical, Co. (St. Louis, MO, USA). All starting materials were obtained from commercial sources (Merck, Merck KGaA, Darmstadt, Germany, Fluka Sigma-Aldrich Laborchemikalien GmbH, Hannover, Germany, Alfa Aesar, Karlsruhe, Germany, and Sigma, St. Louis, MO, USA) and used without further purification. Melting points (uncorrected) were determined on a MEL-Temp II (Lab. Devices, Holliston, MA, USA). For the in vitro tests, UV–VIS spectra were obtained on a 554 double-beam spectrophotometer Perkin-Elmer (Perkin-Elmer Corporation Ltd., Lane Beaconsfield, Bucks, UK). Infrared spectra (KBr pellets) were recorded with Perkin-Elmer 597 spectrophotometer (Perkin-Elmer Corporation Ltd., Lane Beaconsfield, Bucks, UK). The 1H Nucleic Magnetic Resonance (NMR) spectra were recorded at 300 MHz on a Bruker AM-300 spectrometer (Bruker Analytische Messtechnik GmbH, Rheinstetten, Germany) in CDCl3 or DMSO using tetramethylsilane as an internal standard unless otherwise stated. 13C-NMR spectra were obtained at 75.5 MHz on a Bruker AM-300 spectrometer (Bruker, Hamburg, Germany) in CDCl3 or DMSO solutions with tetramethylsilane as internal reference unless otherwise stated. Chemical shifts are expressed in ppm and coupling constants J in Hz. Mass spectra were determined on a LC-MS 2010 EV Shimadzu (Shimadzu, Kiyoto, Japan) using MeOH as the solvent. Elemental analyses for C and H gave values acceptably close to the theoretical values (±0.4%) in a Perkin-Elmer 240B CHN analyzer (Perkin-Elmer Corporation Ltd., Lane Beaconsfield, Bucks, UK). Reactions were monitored by thin layer chromatography on 5554 F254 silica gel/TLC cards (Merck and Fluka Chemie GmbH Buchs, Steinheim, Switzerland). For preparative thin layer chromatography (prep TLC) silica gel 60 F254, plates 2 mm, Merck KGaA ICH078057 were used. For the experimental determination of the lipophilicity using reverse phase thin layer chromatography (RP-TLC) TLC-Silica gel 60 F254 DC Kieselgel, Merck (Merck, Merck KGaA, Darmstadt, Germany) (20 × 20 cm) plates were used.

### 3.2. Chemistry General Procedure

#### 3.2.1. Synthesis of 4-Hydroxy-Chalcones (**a1**–**13**)

A modified Claisen–Schmidt condensation was performed between 4-hydroxy acetophenone and the suitable substituted aryl aldehyde at a molar ratio 1:1 in absolute ethanol (10 mL) [29]. Three milliliters (3 mL) aqueous KOH (20%) was added. The mixture was stirred at room temperature in a US-bath. The end of the reaction was monitored by TLC. The mixture was treated with aqueous HCl 10% and adjusted to acidic pH. The precipitate was either filtered and washed with cold water or extracted with CHCl_3_ (30 mL × 3). The combined organic layers were washed with water and brine and dried under anhydrous MgSO_4_. The product was evaporated to dryness and purified by recrystallization from a proper solvent.

*(E)-3-(4-((4-bromobenzyl) oxy) phenyl)-1-(4-hydroxyphenyl) prop-2-en-1-one* (**a1**) [29]. The crude product was recrystallized from ethanol: yield: 76%; yellow solid; R_f_ (hexane:acetone 2:1): 0.47; m.p.: 117–119 °C.

*(E)-1-(4-hydroxyphenyl)-3-(3-phenoxyphenyl) prop-2-en-1-one* (**a2**) [29]. The mixture was heated in a microwave oven for 15 min (50 watt, 60 °C). The crude product was recrystallized from ethanol: yield: 69%; yellow solid; R_f_ (hexane:acetone 2:1): 0.5; m.p.: 193–195 °C.

*(2E,4E)-5-(4-(dimethylamino) phenyl)-1-(4-hydroxyphenyl) penta-2,4-dien-1-one* (**a3**) [29]. The crude product was crystallized from ethanol: yield: 79%; dark brown solid; R_f_ (hexane:acetone 2:1): 0.55, 0, 55; m.p.: 220–222 °C.

*(E)-1-(4-hydroxyphenyl)-3-(naphthalen-1-yl) prop-2-en-1-one* (**a4**) [29]. The crude product was recrystallized from PE/EA: yield: 84%; yellow solid; R_f_ (hexane:acetone 2:1): 0.52; m.p.: 148–150 °C.

*(E)-1-(4-hydroxyphenyl)-3-(thiophen-2-yl) prop-2-en-1-one* (**a5**). The crude product was recrystallized from methanol: yield: 96%; yellow solid; R_f_ (hexane:acetone 2:1): 0.41; m.p.: 162–164 °C [67].

*(E)-3-(2,7a-dihydro-1H-indol-3-yl)-1-(4-hydroxyphenyl) prop-2-en-1-one* (**a6**). The crude product was crystallized from ethanol: yield: 77%; R_f_ (hexane:acetone 2:1): 0.36; m.p.: 212–213 °C; IR (KBr, cm^−1^): 1610, 1640; LC-MS (*m*/*z*): (C_17_H_15_NO_2)_ 289 [M + Na]^+^, 298 [M + CH_3_OH]^+^; ^1^H-NMR (300 MHz, CDCl_3_) δ: 10.05 (s, 1H, O*H*−), 8.32 (m, 2H, aromatic protons), 7.85 (m, 1H, aromatic proton), 7.41–7.53 (m, 4H, aromatic protons, =C*H*), 7.29–7.37 (m, 3H, aromatic protons), 6.86–7.14 (m, 3H, aromatic protons, −CO–C*H*=); ^13^C-NMR (75 MHz, CDCl_3_) δ: 184.30 (C=O), 161.87 (C–O−), 143.92 (CH–C=O), 135.82, 124.51, 123.06, 122.02, 111.46, 106.92, 77.19, 76.91, 76.59. Elemental analysis: expected % (C_17_H_15_NO_2_): C 76.96, H 5.7, N 5.28; found % (C_17_H_15_NO_2_): C 76.73, H 5.9, N 5.43.

*(2E,4E)-1-(4-hydroxyphenyl)-5-phenylpenta-2,4-dien-1-one* (**a7**). Methanol was used as a solvent. The mixture was heated at 35 °C in US-bath. The crude product was recrystallized from ethanol: yield: 78%; R_f_ (hexane:acetone 2:1): 0.46; m.p.: 164–166 °C [68].

*(2E,4E)-1-(4-hydroxyphenyl)-4-methyl-5-phenylpenta-2,4-dien-1-one* (**a8**). Methanol was used as a solvent. The crude product was recrystallized from ethanol and treated with diethylether. Yield: 74%; R_f_ (hexane:acetone 2:1): 0.42; m.p.: 100–102 °C; IR (KBr, cm^−1^): 1610,1640; LC-MS (*m*/*z*): (C_18_H_16_O_2_) 265 [M + 1]^+^, 306 [M + CH3CN + H]^+^; ^1^H-NMR (300 MHz, CDCl_3_) δ: 7.90–8.04 (m, 2H, aromatic protons), 7.81 (d, 1H, C*H*=, *J* = 15 Hz), 7.41(d, 1H, C*H*=, *J* = 15 Hz), 7.17–7.31 (m, 6H, aromatic protons), 6.83–6.86 (m, 2H, aromatic protons), 2.47 (s, 3H, CH_3_); ^13^C-NMR (75 MHz, CDCl_3_) δ: 193.1 (C=O), 163.1 (C–O−), 146.1, 145.2, 142.1, 134.2, 131.9, 129.8, 127.4, 125.2, 124,9, 116.4, 15.7 (CH_3_). elemental analysis: expected % (C_18_H_16_O_2_): C 81.79, H 6.10; found % (C_18_H_16_O_2_): C 81.67, H 6.04.

*(2E,4E)-4-bromo-1-(4-hydroxyphenyl)-5-phenylpenta-2,4-dien-1-one* (**a9**). Methanol was used as asolvent. The mixture was heated at 35 °C in an ultrasound bath. The crude product was recrystallized from ethanol: yield: 73%; yellow semi-solid; R_f_ (hexane:acetone 2:1): 0.42; m.p.: 88–89 °C; IR (KBr, cm^−1^): 1610,1650; LC-MS (*m*/*z*): (C_17_H_13_BrO_2_) 363 [M + CH_3_OH + H]^+^; ^1^H-NMR (300 MHz, CDCl_3_) δ: 7.89–8.04 (m, 3H, aromatic protons), 7.77–7.80 (m, 1H, =C*H*–CO), 7.60–7.66 (m, 1H, aromatic proton), 7.36–7.93 (m, 3H, aromatic protons and =C*H*−), 7.02–7.09 (m, 1H, C*H*=C (Br)), 6.88–7.01 (m, 3H, aromatic protons); ^13^C-NMR (75 MHz, CDCl_3_) δ: 196.73 (C=O), 162.59 (C–OH), 130.83, 130.59, 130.43, 129.50 (C–C=O), 128.90 (Ph–C=), 121.83 (C–Br), 114.32, 114.22, 114.12. Elemental analysis: expected % (C_17_H_13_BrO_2_): C 62.03, H 3.98; found % (C_17_H_13_BrO_2_): C 59.97, H 4.18.

*(E)-3-(furan-2-yl)-1-(4-hydroxyphenyl) prop-2-en-1-one* (**a10**). The crude product was recrystallized from ethanol: yield: 81%; light yellow solid; R_f_ (hexane:acetone 2:1): 0.4; m.p.: 153–155 °C [69].

*(2E,4E)-1-(4-hydroxyphenyl)-5-(4-nitrophenyl) penta-2,4-dien-1-one* (**a11**). The crude product was recrystallized from ethanol: yield: 82%; dark brown solid; R_f_ (hexane:acetone 2:1): 0.2; m.p.: 182–183 °C; IR (KBr, cm^−1^): 1620,1710; LC-MS (*m*/*z*): (C_17_H_13_NO_4_) 297 [M + 1]^+^, 342 [M + CH_3_CH_2_OH]^+^, 337 [M + CH_3_CN]^+^; ^1^H-NMR (300 MHz, CDCl_3_) δ: 8.12–8.13 (m, 2H, aromatic protons), 7.99 (m, 2H, aromatic protons), 7.51–7.59 (m, 4H aromatic and C*H*= protons), 7.36–7.38 (m, 2H aromatic protons), 7.02–7.09 (m, 2H aromatic and C*H*=, protons), 6.92–6.93(m, 2H, aromatic protons); ^13^C-NMR (75 MHz, CDCl_3_) δ: 192.80 (C=O), 162.74 (C–OH), 146.50, 146.12, 141.97, 136.42, 129.40, 127.93, 127.54 (CH–CO), 125.83, 124.70, 116.63. Elemental analysis: expected % (C_17_H_13_NO_4_): C 69.15, H 4.44, N 4.74; found % (C_17_H_13_NO_4_): C 69.25, H 4.21, N 5.01.

*(E)-1-(4-hydroxyphenyl)-3-(5-methylfuran-2-yl) prop-2-en-1-one* (**a12**). The crude product was recrystallized from ethanol: yield: 89%; orange-yellow solid; R_f_ (hexane:acetone 2:1): 0.44; m.p.: 157–159 °C; IR (KBr, cm^−1^): 1610, 1640; ^1^H-NMR (300 MHz, CDCl_3_) δ: 8.01–8.05 (m, 2H, aromatic protons), 7.52 (d, 1H, C*H*=, *J* = 15 Hz), 7.36 (d, 1H, C*H*=, *J* = 15 Hz), 6.92–6.66 (m, 4H, aromatic protons), 5.77 (s, 1H, O*H*), 2.39 (s, 3H, CH_3_); ^13^C-NMR (75 MHz, CDCl_3_) δ: 188.37 (C=O), 160.04, 150.42, 130.99, 130.38, 117.94, 117.39, 115.35, 114.93, 109.70, 109.21, 23.04 (−CH_2_−), 14.02 (CH_3_); elemental analysis: expected % (C_14_H_12_O_3_): C 77.89, H 5.03; found % (C_13_H_12_O_2_): C 78.1, H 5.68.

*(E)-1-(4-hydroxyphenyl)-3-phenylprop-2-en-1-one* (**a13**). The crude product was crystallized from ethanol: yield: 74%; white solid; R_f_ (hexane:acetone 2:1): 0.48; m.p.: 122–123 °C [70].

#### 3.2.2. Synthesis of 2-Hydroxy-Chalcones (**b1**–**4, b7**–**9, b11, b13**)

A modified Claisen–Schmidt condensation was performed between 2-hydroxy acetophenone and the suitable substituted aryl aldehyde at a molar ratio 1:1 in absolute ethanol (10 mL). Three milliliters (3 mL) of aqueous KOH (20%) was added. The mixture was stirred at room temperature in a US-bath. The end of the reaction was monitored by TLC. The mixture was treated with aqueous HCl 10% and adjusted to acidic pH. The precipitate was either filtered and washed with cold water or extracted with CHCl_3_ (30 mL × 3). The combined organic layers were washed with water and brine and dried under anhydrous MgSO_4_. The product was evaporated to dryness and purified by recrystallization from a proper solvent.

*(E)-3-(4-((4-bromobenzyl) oxy) phenyl)-1-(2-hydroxyphenyl) prop-2-en-1-one* (**b1**). The crude product was crystallized from acetone: yield: 70%; bright yellow solid; R_f_ (dichloromethane): 0.8; m.p.: 76 °C; IR (KBr, cm^−1^): 3060, 3100,1680, 1580, 1100; ^1^H-NMR (300 MHz, CDCl_3_) δ: 8.06–8.18 (s, 1H, OH), 6.91–7.92 (m, 14H, aromatic and =C*H*–CO protons), 5.17 (s, 2H, −OCH_2_); ^13^C-NMR (75 MHz, CDCl_3_) δ: 193.62 (C=O), 163.57, 163.37 (C–OH), 160.82, 145.09, 136.00, 135.40, 132.00, 131.86, 130.54, 130.31, 129.05, 127.82, 122.30 (C–Br), 120.10, 118.76, 118.60, 118.00, 115.36, 115.10, 77.42, 77.00, 76.57, 69.46 (−CH_2_–O−); elemental analysis: expected % (C_22_H_17_BrO_3_): C 64.56, H 4.19; found % (C_22_H_17_BrO_3_): C 54.44, H 4.02.

*(E)-1-(2-hydroxyphenyl)-3-(3-phenoxyphenyl) prop-2-en-1-one* (**b2**). The reaction was assisted with microwave at 65 °C, 50 watt for 15 min. The crude product was recrystallized from ethanol: yield: 68%; yellow solid; R_f_ (dichloromethane): 0.8; m.p.: 76 °C; IR (KBr, cm^−1^): 3100,1700, 3050; ^1^H-NMR (300 MHz, CDCl_3_) δ: 12.90 (s, 1H, OH), 7.82–7.90 (m, 7H aromatic and C*H*= protons), 7.67–7.7.72 (br, 2H aromatic protons), 7.41–7.54 (m, 3H aromatic protons), 6.91–7.18 (m, 3H, aromatic and C*H*=, protons); ^13^C-NMR (75 MHz, CDCl_3_) δ: 187.21 (C=O), 163.60, 163.37, 160.82, 144.84, 141.89, 132.64, 129.21, 128.82, 128.56, 127.27, 126.95, 125.43, 77.42; elemental analysis: expected % (C_21_H_16_O_3_): C 79.73, H 4.78; found % (C_21_H_16_O_3_): C 79.76, H 4.39.

*(2E,4E)-5-(4-(dimethylamino)phenyl)-1-(2-hydroxyphenyl)penta-2,4-dien-1-one* (**b3**) The crude product was crystallized from methanol/chloroform Yield: 71%; Dark purple semi-solid; R_f_ (dichloromethane): 0.9; m.p.: semi-solid; IR (KBr, cm^−1^): 3100, 3060, 1720, 1580, 1300; ^1^H-NMR (300 MHz, CDCl_3_) δ: 12.28 (s, 1H, OH), 7.67–7.7.72 (br, 2H aromatic protons), 7.41–7.54 (m, 3H aromatic protons), 6.91–7.18 (m, 3H, aromatic and C*H*=, protons) 6.50–6.85 (m, 4H aromatic and C*H*=, protons), 2.63–3.27 (m, 6H, 2 × CH_3_); ^13^C-NMR (75 MHz, CDCl_3_) δ: 190.28 (C=O), 153.89, 152.37, 136.40, 132.00, 130.67, 130.44, 123.75, 121.74, 118.86, 118.4, 117.97, 111.71, 110.94, 110.61, 77.40, 77.00, 76.54, 40.00 (CH_3_); elemental analysis: expected % (C_19_H_19_NO_2_): C 77.79, H 6.53, N 4.77; found % (C_19_H_19_NO_2_): C 77.56, H 6.14, N 4.84.

*(E)-1-(2-hydroxyphenyl)-3-(naphthalen-1-yl) prop-2-en-1-one* (**b4**). The crude product was recrystallized with flash chromatography hexane/acetone (2:1): yield: 68%; yellow solid; R_f_ (dichloromethane): 0.9; m.p.: 84–85 °C [71].

*(2E,4E)-1-(2-hydroxyphenyl)-5-phenylpenta-2,4-dien-1-one* (**b7**). Methanol was used as a solvent. The mixture was heated at 35 °C in an ultrasound bath. The crude product was recrystallized from acetone: yield: 72%; bright yellow solid; R_f_ (hexane:acetone 2:1): 0.48; m.p.: 82–83 °C [72].

*(2E,4E)-1-(2-hydroxyphenyl)-4-methyl-5-phenylpenta-2,4-dien-1-one* (**b8**). Methanol was used as solvent. The mixture was heated at 35 °C in an ultrasound bath. The crude product was crystallized from acetone: yield: 67%; yellow semi-solid; R_f_ (hexane:acetone 2:1): 0.68; m.p.: semi-solid; IR (KBr, cm^−1^): 3150, 1620; LC-MS (*m*/*z*): (C_19_H_16_O_2_) 266 [M + 1]^+^, 297 [M + CH_3_OH]^+^, 288 [M + Na]^+^, ^1^H-NMR (300 MHz, CDCl_3_) δ: 7.97–7.99 (m, 2H, C*H*=C), 7.61–7.62 (m, 4H aromatic protons), 7.12–7.37 (m, 3H, aromatic protons, =C*H*–CO), 6.92–7.02 (m, 2H, aromatic protons), 6.4–6.69 (m, 2H, aromatic proton, Ph–C*H*=), 2.02 (s, 3H, CH_3_); ^13^C-NMR (75 MHz, CDCl_3_) δ: 196.97 (C=O), 162.73 (C–OH), 132.02 (−C*H*–C=O), 131.82, 130.88, 130.45, 127.97, 127.70, 123.40, 119.25, 118.74, 116.28, 16.20 (CH_3_); elemental analysis: expected % (C_19_H_16_O_2_): C 81.79, H 6.10; found % (C_19_H_16_O_2_): C 82.01, H 5.99.

*(2E,4E)-4-bromo-1-(2-hydroxyphenyl)-5-phenylpenta-2,4-dien-1-one* (**b9**). Methanol was used as a solvent. The mixture was heated at 35 °C in an ultrasound bath. The crude product was crystallized from ethanol: yield: 65%; yellow semi-solid; R_f_ (hexane:acetone 2:1): 0.48; m.p.: semi-solid; IR (KBr, cm^−1^): 3150, 1690, 1610; LC-MS (*m*/*z*): (C_17_H_13_BrO_2_) 331 [M + 1]^+^, 376 [M + CH_3_CH_2_OH]^+^; ^1^H-NMR (300 MHz, CDCl_3_) δ: 7.89–8.04 (m, 4H, aromatic and OH, protons), 7.80 (d, 1H, C*H*=, *J* = 15 Hz), 7.60–7.66 (m, 1H), 7.54 (d, 1H, C*H*=, *J* = 15 Hz), 7.36–7.44 (m, 2H), 7.02–7.09 (m, 1H), 6.89–7.01 (m, 3H, aromatic protons); ^13^C-NMR (75 MHz, CDCl_3_) δ: 196.73 (C=O), 162.59 (C–OH), 130.83, 130.71, 130.59, 130.43, 129.50, 128.90, 128.30, 121.83, 114.32, 114.22, 114.12 (C–Br); elemental analysis: expected % (C_17_H_13_BrO_2_): C 62.03, H 3.98; found % (C_17_H_13_BrO_2_): C62.33, H 4.15.

*(2E,4E)-1-(2-hydroxyphenyl)-5-(4-nitrophenyl) penta-2,4-dien-1-one* (**b11**). The crude product was crystallized from methanol: yield: 76%; R_f_ (hexane:acetone 2:1): 0.65; m.p.: 146–148 °C; IR (KBr, cm^−1^): 3200, 1710,1620; LC-MS (*m*/*z*): (C_17_H_13_NO_4_) 297 [M + 1]^+^, 342 [M + CH_3_CH_2_OH]^+^, 307 [M + CH_3_CN]^+^; ^1^H-NMR (300 MHz, CDCl3) δ: 8.12–8.13 (m, 1H, OH proton), 7.99–8.01 (m, 2H, aromatic proton), 7.51–7.59 (m, 4H, aromatic and C*H*=, protons), 7.36–7.38 (m, 2H aromatic protons), 7.02–7.09 (m, 2H aromatic protons), 6.92–6.93 (m, 2H aromatic and C*H*=, protons); ^13^C-NMR (75 MHz, CDCl3) δ: 191.97 (C=O), 163.40 (C–OH), 146.45 (C–NO_2_), 146.12 (−C=C−), 141.97, 135.90, 129.67, 127.93, 127.54, 125.83, 124.70, 117.90; elemental analysis: expected % (C_17_H_13_NO_4_): C 69.15, H 4.44, N 4.74; found % (C_17_H_13_NO_4_): C 69.36, H 4.72, N 4.86.

*(E)-1-(2-hydroxyphenyl)-3-phenylprop-2-en-1-one* (**b13**). The crude product was recrystallized from ethanol: yield: 78%; R_f_ (hexane:acetone 2:1): 0.56; m.p.: 75–78 °C [73].

#### 3.2.3. Synthesis of *bis*-Ethers (**di**–**dvii**)

4-hydroxy acetophenone and 1, 3-dibromoalkane in molar ratio 2:1 was diluted in 50 mL acetone. Anhydrous K_2_CO_3_ was added and the mixture was refluxed for approximately 18 h. the reaction was completed judged by the negative alcoholic ferric chloride (3%) test. The mixture was evaporated to dryness. The solid was treated with water, filtered of and recrystallized from absolute ethanol [28].

*1,1′-((ethane-1,2-diylbis(oxy))bis(4,1-phenylene))diethanone* (**di**) [74]. The crude product was recrystallized from ethanol: yield: 92%; white crystals; R_f_ (hexane:acetone 2:1): 0.4; m.p.: 161 °C.

*1,1′-((propane-1, 3-diyl bis-(oxy))bis(4,1-phenylene))diethanone* (**dii**). The crude product was crystallized from ethanol: yield: 90%; white crystals; R_f_ (hexane:acetone 2:1): 0.53; m.p.: 115–117 °C [29].

*1,1′-((butane-1,4-diylbis(oxy))bis(4,1-phenylene))diethanone* (**diii**) [43]. The crude product was crystallized from ethanol: yield: 87%; white crystals; R_f_ (hexane:acetone 2:1): 0.42; m.p.: 142 °C [74].

*1,1′-((pentane-1,5-diylbis(oxy))bis(4,1-phenylene))diethanone* (**div**). The crude product was crystallized from ethanol: yield: 82%; white crystals; R_f_ (hexane:acetone 2:1): 0.41; m.p.: 87 °C; IR (KBr, cm^−1^): 1620, 1700, 3040; ^1^H-NMR (300 MHz, CDCl_3_) δ: 7.90–7.91 (d, 4H, aromatic protons), 6.89–6.91 (m, 4H, aromatic protons), 4.02–4.05 (m, 4H, −CH_2_–O), 3.68–3.72 (m, 2H, −CH_2_−), 2.53 (s, 6H, 2 × CH_3_), 1.85–1.92 (m, 4H, −CH_2_−); ^13^C-NMR (75 MHz, CDCl_3_) δ: 196.78 (C=O), 162.93 (C–O−), 87.90, 77.30, 77.04, 76.79 (−C–CH_2_–O), 28.87, 26.36, 22.56 (−CH_2_−); elemental analysis: expected % (C_21_H_24_O_4_): C 74.09, H 7.11; found % (C_21_H_24_O_4_): C 74.05, H 6.94.

*1,1′-((hexane-1,6-diylbis(oxy))bis(4,1-phenylene))diethanone* (**dv**) [74]. The crude product was crystallized from ethanol: yield: 89%; white crystals; Rf (hexane:acetone 2:1): 0.42; m.p.: 121 °C.

*1,1′-((heptane-1,7-diylbis(oxy)) bis-(4,1-phenylene)) diethanone* (**dvi**). The crude product was crystallized from ethanol: yield: 94%; white crystals; R_f_ (hexane:acetone 2:1): 0.45; m.p.: 111 °C; IR (KBr, cm^−1^): 1610, 1700, 3030; ^1^H-NMR (300 MHz, CDCl_3_) δ: 7.92–8.00 (m, 4H, aromaticand C*H*=, protons), 6.88–6.90 (m, 4H, aromatic and C*H*=, protons), 4.29–4.31 (m, 4H, CH_2_–O), 2.56 (s, 6H, 2 × CH_3_), 1.86–1.91 (m, 4H, aliphatic protons), 1.44–1.48 (m, 4H, aliphatic protons), 1.31–1.32 (m, 2H, −CH_2_−); ^13^C-NMR (75 MHz, CDCl_3_) δ: 197.15 (C=O), 196.70, 165.40, 163.25 (C–O), 130.04, 128.35, 127.80, 67.43, 30.24, 23.01 (CH_3_), 22.65 (−CH_2_−); elemental analysis: expected % (C_23_H_28_O_4_): C 74.97, H 7.66; found % (C_23_H_28_O_4_): C 74.94, H 7.61.

*1,1′-((octane-1,8-diylbis(oxy)) bis-(4,1-phenylene))diethanone* (**dvii**). The crude product was crystallized from ethanol: yield: 93%; white crystals; R_f_ (hexane:acetone 2:1): 0.46; m.p.: 123 °C; IR (KBr, cm^−1^): 1610, 1690, 3040; ^1^H-NMR (300 MHz, CDCl_3_) δ: 7.98–8.07 (m, 4H, aromatic and C*H*=, protons), 6.95–7.02 (m, 4H, aromatic and C*H*=, protons), 4.38–4.40 (m, 4H, aliphatic protons), 2.53 (s, 6H, 2 × CH_3_), 1.98–2.01 (m, 4H, aliphatic protons), 1.45–1.49 (m, 4H, aliphatic protons), 1.29–1.30 (m, 4H, aliphatic protons); ^13^C-NMR (75 MHz, CDCl_3_) δ: 196.80 (C=O), 162.95 (−C–O−), 129.34, 128.70, 127.35, 67.83 (−CH_2_–O−), 29.74, 28.80, 23.62; elemental analysis: expected % (C_24_H_30_O_4_): C 75.36, H 7.91; found % (C_24_H_30_O_4_): C 75.31, H 7.88.

#### 3.2.4. Synthesis of *bis*-Etherified Double Chalcones (**c1**–**13**)

A Claisen–Schmidt condensation was performed between (1,1′-((propane-1,3-diyl-*bis*(oxy)) *bis*-(4,1-phenylene))diethanone) and the appropriate substituted aromatic aldehyde at a molar ratio 1:2 in absolute ethanol (10 mL). Three milliliter (3 mL) aqueous KOH (20%) was added. The mixture was stirred at room temperature in a US-bath. The end of the reaction was monitored by TLC. After the completion of the reaction the mixture was treated with aqueous HCl 10% and adjusted to acidic pH. The precipitate was either filtered and washed with cold water or extracted with CHCl_3_ (30 mL × 3). The combined organic layers were washed with water and brine and dried under anhydrous MgSO_4_. The product was evaporated to dryness and purified by recrystallization from a proper solvent.

*(2E,2′E)-1,1′-((propane-1,3-diylbis(oxy))bis(4,1-phenylene))bis(3-(4-((4-bromobenzyl)oxy)phenyl)prop-2-en-1-one)* (**c1**). The crude product was crystallized from acetone: yield: 89%; yellow solid; R_f_ (hexane:acetone 2:1): 0.5; m.p.: 197–199 °C [29].

*(2E,2′E)-1,1′-((propane-1,3-diylbis(oxy))bis(4,1-phenylene))bis(3-(3-phenoxyphenyl)prop-2-en-1-one*) (**c2**). The crude product was crystallized from ethanol: yield: 72%; yellow solid; R_f_ (hexane:acetone 2:1): 0.68; m.p.: 172–174 °C [29].

*(2E,2′E, 4E, 4′E)-1,1′-((propane-1,3-diylbis-(oxy)) bis(4,1-phenylene)) bis-(5-(4-(dimethylamino) phenyl) penta-2,4-dien-1-one)* (**c3**). The crude product was recrystallized from ethanol: yield: 79%; Dark brown solid; R_f_ (hexane:acetone 2:1): 0.55, 0.62; m.p.: 156–158 °C [29].

*(2E,2′E)-1, 1′-((propane-1,3-diylbis-(oxy)) bis-(4,1-phenylene)) bis-(3-(naphthalen-1-yl) prop-2-en-1-one)* (**c4**). The crude product was recrystallized from PE/EA: yield: 74%; yellow solid; R_f_ (hexane:acetone 2:1): 0.64; m.p.: 167–168 °C [29].

*(2E,2′E)-1,1′-((propane-1,3-diyl bis-(oxy)) bis-(4,1-phenylene)) bis-(3-(thiophen-2-yl) prop-2-en-1-one)* (**c5**). The crude product was recrystallized with ethanol: yield: 83%; light yellow solid; R_f_ (hexane:acetone 2:1): 0.4; m.p.: 132 °C; IR (KBr, cm^−1^): 1610, 1650; ^1^H-NMR (300 MHz, CDCl_3_) δ: 8.27–8.25 (m, 4H, phenyl aromatic and heteroaromatic protons −CH–S), 7.93–7.98 (m, 4H, aromatic and C*H*= protons), 7.28–7.34 (m, 6H, aromatic protons,), 7.05–7.14 (m, 2H, aromatic protons), 6.91–7.03 (m, 6H, aromatic and C*H*= protons), 4.19–4.29 (m, 4H, aliphatic protons), 2.30–2.36 (m, 2H, aliphatic protons); ^13^C-NMR (75 MHz, CDCl_3_) δ: 187.02 (C=O), 162.64 (−C–O–), 139.45 (−C–S), 135.24, 132.01, 130.89, 130.53, 128.92, 127.54, 120.87, 115.01, 67.42 (−CH_2_–O−), 24.90 (−CH_2_−); elemental analysis: expected % (C_29_H_24_O_4_S_2_): C 67.8, H 4.38; found % (C_29_H_24_O_4_S_2_): C 67.6, H 4.18.

*(2E,2′E)-1,1′-((propane-1,3-diylbis(oxy))bis(4,1-phenylene))bis(3-(1H-indol-3-yl)prop-2-en-1-one)* (**c6**). The crude product was recrystallized with ethanol: yield: 81%; white solid; R_f_ (hexane:acetone 2:1): 0.42; m.p.: 197 °C; IR (KBr, cm^−1^): 1610, 1640; LC-MS (*m*/*z*): (C_37_H_30_N_2_O_4_) 606 [M + K]^+^, 640 [M + CH_3_CN + Na]^+^; ^1^H-NMR (300 MHz, CDCl_3_) δ: 10.05 (br, 2H, −NH), 7.87–7.93 (m, 4H, aromatic protons), 7.67–7.79 (m, 4H, aromatic and C*H*= protons), 7.39–7.44 (m, 2H aromatic protons), 7.16–7.28 (m, 6H aromatic protons), 6.99–7.11 (m, 6H aromatic and C*H*= protons), 4.21–4.27 (m, 4H, aliphatic protons), 2.28–2.35 (m, 2H, aliphatic protons); ^13^C-NMR (75 MHz, CDCl_3_) δ: 191.63 (C=O), 163.24 (−C–O), 145.53, 129.92, 126.37, 118.16, 117.82, 114.40, 113.20, 112.73, 64.92 (C–N), 28.70 (−CH_2_−); elemental analysis: expected % (C_37_H_30_N_2_O_4_): C 82.20, H 5.97; found % (C_37_H_30_N_2_O_4_): C 82.0, H 5.71.

*(2E,2′E,4E,4′E)-1,1′-((propane-1,3-diylbis(oxy))bis(4,1-phenylene))bis(5-phenylpenta-2,4-dien-1-one)* (**c7**). The crude product was recrystallized with flash chromatography hexane/acetone (2:1): yield: 74%; orange-yellow solid; R_f_ (hexane:acetone 2:1): 0.9; m.p.: 98–100 °C; IR (KBr, cm^−1^): 1600, 1650, 1710; LC-MS (*m*/*z*): (C_37_H_32_O_4_) 542 [M + 1]^+^=, 587 [M + CH_3_CH_2_OH]^+^, 540[M − 1]^+^; ^1^H-NMR (300 MHz, CDCl_3_) δ: 7.88–8.06 (m, 8H, aromatic protons), 7.79 (d, 2H, C*H*=, *J* = 15 Hz), 7.53–7.66 (m, 2H, aromatic protons), 7.47–7.53 (m, 2H, aromatic protons), 7.30–7.43 (br, 4H, aromatic protons), 7.10 (d, 2H, =C*H*, *J* = 15 Hz), 6.90–7.03 (m, 8H aromatic protons), 4.22–4.27 (m, 4H, aliphatic protons), 2.29–2.36 (m, 2H, aliphatic protons); ^13^C-NMR (75 MHz, CDCl_3_) δ: 190.12 (C=O), 163.77 (−C–O), 143.97 (−C*H*=C*H*−), 131.04, 128.82, 127.60, 124.72, 116.25, 64.60, 29.42 (−CH_2_−); elemental analysis: expected % (C_37_H_32_O_4_): C 82.20, H 5.97; found % (C_37_H_32_O_4_): C 82.0, H 5.71.

*(2E,2′E,4E,4′E)-1,1′-((propane-1,3-diylbis(oxy))bis(4,1-phenylene))bis(4-methyl-5-phenylpenta-2,4-dien-1-one)* (**c8**). The crude product was recrystallized with ethanol: yield: 72%; yellow solid; R_f_ (hexane:acetone 2:1): 0.46; m.p.: 90–92 °C; IR (KBr, cm^−1^): 1610, 1720; LC-MS (*m*/*z*): (C_39_H_36_O_4_) 570 [M + 1]^+^, 615 [M + CH_3_CH_2_OH]^+^; ^1^H-NMR (300 MHz, CDCl_3_) δ: 7.98–8.05 (m, 4H, aromatic protons), 7.91–7.95 (m, 4H, aromatic protons), 7.80 (d, 2H, C*H*=, *J* = 15 Hz), 7.51–7.66 (br, 4H aromatic protons), 7.36–7.42 (m, 4H, aromatic protons), 7.05 (d, 2H, =C*H*, *J* = 15 Hz), 6.92–7.00 (m, 8H, aromatic protons), 4.21–4.27 (m, 4H, aliphatic protons), 2.55 (s, 6H, 2 × CH_3_), 2.29–2.36 (m, 2H, aliphatic protons); ^13^C-NMR (75 MHz, CDCl_3_) δ: 191.02 (C=O), 164.78 (C–O), 143.97, 129.84, 128.82, 127.60, 124.72, 115.20, 64.60, 29.42 (−CH_2_−); elemental analysis: expected % (C_39_H_36_O_4_): C 82.20, H 5.97; found % (C_39_H_36_O_4_): C 82.0, H 5.71.

*(2E,2′E,4E,4′E)-1,1′-((propane-1,3-diylbis(oxy))bis(4,1-phenylene))bis(4-bromo-5-phenylpenta-2,4-dien-1-one)* (**c9**). The crude product was recrystallized with ethanol: yield: 70%; light yellow solid; R_f_ (hexane:acetone 2:1): 0.46; m.p.: 120–122 °C; IR (KBr, cm^−1^): 1610, 1700; LC-MS (*m*/*z*): (C_37_H_30_Br_2_O_4_) 700 [M + 1]^+^, 745 [M + CH_3_CH_2_OH]^+^; ^1^H-NMR (300 MHz, CDCl_3_) δ: 7.83–7.94 (br, 4H, aromatic and C*H*= protons), 7.46–7.68 (m, 6H, aromatic protons), 7.15–7.28 (m, 6H, aromatic protons), 6.74–6.92 (m, 8H, aromatic and =C*H* protons), 4.20–4.24 (m, 4H, aliphatic protons), 2.34–2.39 (m, 2H, aliphatic protons); ^13^C-NMR (75 MHz, CDCl_3_,) δ: 189.60 (C=O), 164.70 (−C–O), 143.97, 133.25, 131.04, 128.82, 127.60, 124.72, 122.01, 116.25 (C–Br), 115.07, 64.60, 29.13 (−CH_2_−); elemental analysis: expected % (C_37_H_30_Br_2_O_4_): C 63.63, H 4.33; found % (C_37_H_30_Br_2_O_4_): C63.78, H 4.55. 

*(2E,2′E)-1,1′-((propane-1,3-diylbis(oxy))bis(4,1-phenylene))bis(3-(furan-2-yl)prop-2-en-1-one)* (**c10**). The crude product was crystallized with PE/EA Yield: 89%; Light yellow solid; R_f_ (hexane:acetone 2:1): 0.42; m.p.: 159–160 °C; IR (KBr, cm^−1^): 1610,1640,1730; LC-MS (*m*/*z*): (C_29_H_24_O_6_) 470 [M + 1]^+^, 533 [M + CH_3_CN + Na]^+^; ^1^H-NMR (300 MHz, CDCl_3_,) δ: 7.95–8.03 (m, 4H, aromatic and C*H*= protons), 7.41–7.62 (m, 6H), 6.92–6.98 (m, 4H, aromatic and =C*H* protons), 6.66–6.71 (br, 2H), 6.48–6.53 (br, 2H), 4.21–4.29 (m, 4H, aliphatic protons), 2.28–2.38 (m, 2H, aliphatic protons); ^13^C-NMR (75 MHz, CDCl_3_,) δ: 188.08 (C=O), 162.81 (−C–O), 144.74 (−CH_2_–C–O), 131.60, 131.32, 130.46, 129.89, 119.08, 116.24, 114.59, 112.31, 64.34, 29.41 (−CH_2_−); elemental analysis: expected % (C_29_H_24_O_6_): C 74.35, H 5.16; found % (C_29_H_24_O_6_): C 74.43, H 4.77.

*(2E,2′E,4E,4′E)-1,1′-((propane-1,3-diylbis(oxy))bis(4,1-phenylene))bis(5-(4-nitrophenyl)penta-2,4-dien-1-one)* (**c11**). The crude product was recrystallized with ethanol: yield: 87%; dDark brown solid; R_f_ (hexane:acetone 2:1): 0.34; m.p.: 278–280 °C; IR (KBr, cm^−1^): 1620, 1730; LC-MS (*m*/*z*): (C_37_H_30_N_2_O_8_) 630 [M − 1]^+^, 654 [M + Na]^+^; ^1^H-NMR (300 MHz, CDCl_3_,) δ: 8.21–8.25 (m, 4H, aromatic protons), 7.96–8.01 (m, 2H, aromatic and C*H*= protons), 7.90–7.96 (m, 4H), 7.53–7.66 (m, 4H, aromatic protons), 7.10–7.23 (m, 2H), 6.90–7.05 (m, 8H, aromatic and =C*H*, protons), 4.21–4.28 (m, 4H, aliphatic protons), 2.29–2.38 (m, 2H, aliphatic protons); ^13^C-NMR (75 MHz, CDCl_3_) δ: 191.31 (C=O), 164.02 (−C–O), 146.07 (−C–NO_2_), 142.44, 130.72, 126.94, 125.60, 123.42, 121.70, 114.69, 66.01, 26.42 (−CH_2_−); elemental analysis: expected % (C_37_H_30_N_2_O_8_): C 70.47, H 4.79, N 4.44; found % (C_37_H_30_N_2_O_8_): C70.67, H 4.53, N 4.13.

*(2E,2′E)-1,1′-((propane-1,3-diylbis(oxy))bis(4,1-phenylene))bis(3-(5-methylfuran-2-yl)prop-2-en-1-one)* (**c12**). The crude product was recrystallized with PE/EA: yield: 90%; orange-yellow solid; R_f_ (hexane:acetone 2:1): 0.44; m.p.: 107–109 °C; IR (KBr, cm^−1^): 1620, 1680; LC-MS (*m*/*z*): (C_31_H_28_O_6_) 498 [M + 1]^+^, 456 [M − CH_3_CN]^+^; ^1^H-NMR (300 MHz, CDCl_3_) δ: 7.98–8.08 (m, 2H, aromatic protons), 7.86–7.96 (m, 2H aromatic and C*H*= protons), 7.31–7.57 (m, 2H aromatic protons), 6.91–7.17 (m, 6H, aromatic protons), 6.11–6.59 (m, 4H aromatic and =C*H* protons), 4.14–4.32 (m, 4H, aliphatic protons), 2.56 (s, 6H, 2 × CH_3_), 2.28–2.41 (m, 2H, aliphatic protons); ^13^C-NMR (75 MHz, CDCl_3_) δ: 188.28 (C=O), 162.20 (−C–O), 131.49, 130.40, 128.28, 122.28, 118.17, 117.78, 117.08, 113.78, 110.18, 67.27 (−CH_2_–O), 28.47 (−CH_2_−), 14.05 (−CH_3_); elemental analysis: expected % (C_31_H_28_O_6_): C 74.98, H 5.68; found % (C_31_H_28_O_6_): C 74.79, H 5.29.

*(2E,2′E)-1,1′-((propane-1,3-diylbis(oxy))bis(4,1-phenylene))bis(3-phenylprop-2-en-1-one)* (**c13**). The crude product was recrystallized with PE/EA: yield: 81%; white solid; R_f_ (hexane:acetone 2:1): 0.45; m.p.: 148–150 °C; IR (KBr, cm^−1^): 1630,1700; LC-MS (*m*/*z*): (C_33_H_28_O_4_) 490 [M + 1]^+^, 512 [M + Na]^+^; ^1^H-NMR (300 MHz, CDCl_3_) δ: 8.12–8.16 (m, 4H, aromatic protons), 7.76–7.78 (m, 2H aromatic and CH= protons), 7.51–7.59 (m, 6H, aromatic protons), 7.38–7.45 (m, 6H, aromatic protons), 7.06–7.09 (m, 4H, aromatic and C*H*= protons), 4.02–4.07 (m, 4H, aliphatic protons), 2.16–2.21 (m, 2H, aliphatic protons); ^13^C-NMR (75 MHz, CDCl_3_) δ: 190.02 (C=O), 163.54 (−C–O), 144.76 (−C–Ph), 134.18, 127.00, 126.89, 126.42, 119.62, 115.35, 64.92 (−CH_2_–O), 24.87 (−CH_2_−); elemental analysis: expected % (C_33_H_28_O_4_): C 74.98, H 5.68; found % (C_33_H_28_O_4_): C 74.79, H 5.29.

#### 3.2.5. Synthesis of *bis*-Etherified Double Chalcones of (E)-3-(4-(Dimethylamino) Phenyl-Acryl-Aldehyde (**ei**, **eiii–evii**)

A Claisen—Schmidt condensation was performed between (E)-3-(4-(dimethylamino) phenyl) acryl-aldehyde and the appropriate ether at a molar ratio 2:1 in absolute ethanol (10 mL). Three milliliters (3 mL) aqueous KOH (40%) was added. The mixture was stirred at 45 °C in a US-bath. The end of the reaction was monitored by TLC. After the completion of the reaction the mixture was treated with aqueous HCl 25% and adjusted to acidic pH. The precipitate was either filtered and washed with cold water and then extracted with CHCl_3_ (30 mL × 3). The combined organic layers were washed with water and brine and dried under anhydrous MgSO_4_. The product was evaporated to dryness and purified by recrystallization from a proper solvent. Chalcone **eii** has been already synthesized and referred to as **c3** [29].

*(2E,4E)-5-(4-(Dimethylamino)phenyl)-1-(4-(2-(4-((2E,4E)-5-(4-(dimethylamino)phenyl)penta-2,4-dien-1-yl)phenoxy)ethoxy)phenyl)penta-2,4-dien-1-one* (**ei**). The crude product was recrystallized with ethanol: yield: 84%; brown solid; R_f_ (hexane:acetone 2:1): 0.25; m.p.: 101 °C; IR (KBr, cm^−1^): 1610, 1670, 1710; ^1^H-NMR (300 MHz, CDCl_3_) δ: 7.90–8.05 (br, 12H aromatic protons), 7.89 (d, 2H, CH=, J = 15 Hz), 7.40 (d, 2H, =CH, J = 15 Hz), 6.98–7.06 (br, 8H, aromatic protons) 4.34 (s, 4H, aliphatic protons), 2.99–3.09 (br, 12H, 4 × CH_3_); ^13^C-NMR (75 MHz, CDCl_3_) δ: 196.59 (C=O), 162.98 (−C–O), 149.19, 130.78, 130.39, 114.49, 106.58, 98.98, 59.18, 26.67 (−CH_2_−), 16.02 (CH_3_); elemental analysis: expected % (C_40_H_40_N_2_O_4_): C 80,24, H 7.07,N 4.68; found % (C_40_H_40_N_2_O_4_): C 80.02, H 6.98, N 4.34.

*(2E,2′E,4E,4′E)-1,1′-((butane-1,4-diylbis(oxy))bis(4,1-phenylene))bis(5-(4-(dimethylamino)phenyl)penta-2,4-dien-1-one*) (**eiii**). The crude product was recrystallized with ethanol: yield: 85%; brown solid; R_f_ (hexane:acetone 2:1): 0.29; m.p.: 138 °C; IR (KBr, cm^−1^): 1610, 1680; ^1^H-NMR (300 MHz, CDCl_3_) δ: 7.93–8.09 (br, 12H, aromatic and C*H*=, protons), 7.402–7.445 (m, 4H aromatic protons), 6.88–7.08 (br, 8H, aromatic and C*H*=, protons), 4.18–4.23 (m, 4H, aliphatic protons), 2.99–3.15 (br, 12H, 4 × CH_3_), 2.05–2.11 (m, 4H, aliphatic protons); ^13^C-NMR (75 MHz, CDCl_3_) δ: 196.78 (C=O), 162.43 (−C–O), 149.60, 139.54, 122.87, 122.25, 121.63, 112.37, 63.08, 41.33, 26.12 (−CH_2_−); elemental analysis: expected % (C_42_H_42_N_2_O_4_): C 78.72, H 6.92, N 4.37; found % (C_42_H_44_N_2_O_4_): C 78.57, H 6.82, N 4.31.

*(2E,2′E,4E,4′E)-1,1′-((pentane-1,5-diylbis(oxy))bis(4,1-phenylene))bis(5-(4-(dimethylamino)phenyl)penta-2,4-dien-1-one)* (**eiv**). The crude product was recrystallized with ethanol: yield: 80%; brown solid; R_f_ (hexane:acetone 2:1): 0.35; m.p.: 117 °C; IR (KBr, cm^−1^): 1610, 1680, 1710; ^1^H-NMR (300 MHz, CDCl_3_, δ: 7.89–7.99 (m, 12H, aromatic and C*H*= protons), 6.79–6.90 (m, 12H, aromatic and C*H*= protons), 4.23–4.28 (m, 4H, aliphatic protons), 2.835 (s, 12H, 4 × CH_3_), 1.80–1.97 (m, 4H, aliphatic protons), 1.45–1.49 (m, 2H, aliphatic protons); ^13^C-NMR (CDCl_3_, 75 MHz) δ: 196.52 (C=O), 163.65 (−C–O), 149.83, 146.07, 132.15, 128.17, 125.96, 125.02, 113.70, 111.34, 68.90, 41.54, 26.73 (−CH_2_−); elemental analysis: expected % (C_43_H_46_N_2_O_4_): C 78.87, H 7.08, N 4.28; found % (C_43_H_46_N_2_O_4_): C 78.45, H 6.94, N 4.09.

*(2E,2′E,4E,4′E)-1,1′-((hexane-1,6-diylbis(oxy))bis(4,1-phenylene))bis(5-(4-(dimethylamino)phenyl)penta-2,4-dien-1-one)* (**ev**). The crude product was recrystallized with ethanol: yield: 91%; brown solid; R_f_ (hexane:acetone 2:1): 0.43; m.p.: 108 °C; IR (KBr, cm^−1^): 1605, 1710, 1710; ^1^H-NMR (300 MHz, CDCl_3_) δ: 7.88–7.98 (m, 12H, aromatic and C*H*=, protons), 6.88–6.96 (m, 12H, aromatic and C*H*= protons), 4.10–4.19 (m, 4H, aliphatic protons) 2.740–2.99 (br, 12H, 4 × CH_3_), 1.882–1.925 (m, 4H, aliphatic protons), 1.462–1.510 (m, 4H, aliphatic protons); ^13^C-NMR (75 MHz, CDCl_3_,) δ: 196.72 (C=O), 162.94 (−C–O), 130.90, 130.22, 125.58, 118.98, 114.67, 68.21, 39.65 (CH_3_), 28.90, 27.07, 25.48; elemental analysis: expected % (C_44_H_48_N_2_O_4_): C 79.01, H 7.23, N 4.19; found % (C_44_H_48_N_2_O_4_): C 78.91, H 7.19, N 4.14.

*(2E,2′E,4E,4′E)-1,1′-((heptane-1,7-diylbis(oxy))bis(4,1-phenylene))bis(5-(4-(dimethylamino)phenyl)penta-2,4-dien-1-one)* (**evi**). The crude product was recrystallized with ethanol: yield: 90%; brown solid; R_f_ (hexane:acetone 2:1): 0.46; m.p.: 104 °C; IR (KBr, cm^−1^): 1605, 1690; ^1^H-NMR (300 MHz, CDCl_3_) δ: 7.89–7.98 (m, 12H, aromatic and C*H*= protons) 6.88–6.96 (m, 12H, aromatic and C*H*= protons), 4.00–4.05 (m, 4H, aliphatic protons), 2.56–2.89 (br, 12H, 4 × CH_3_), 1.78–1.85 (m, 4H, aliphatic protons) 1.47–1.52 (m, 4H), 1.22–1.26 (m, 2H, aliphatic protons); ^13^C-NMR (75 MHz, CDCl_3_) δ: 196.76 (C=O), 163.04 (−C–O), 151.58, 130.55, 130.12, 114.09, 68.10, 58.39, 40.55, 29.00 (−CH_2_–); elemental analysis: expected % (C_45_H_50_N_2_O_4_): C 79.15, H 7.38, N 4.10; found % (C_45_H_50_N_2_O_4_): C 78.94, H 7.29, N 4.03.

*(2E,2′E,4E,4′E)-1,1′-((octane-1,8-diylbis(oxy))bis(4,1-phenylene))bis(5-(4-(dimethylamino)phenyl)penta-2,4-dien-1-one)* (**evii**). The crude product was recrystallized with ethanol: yield: 93%; brown solid; R_f_ (hexane:acetone 2:1): 0.5; m.p.: 112 °C; IR (KBr, cm^−1^): 1610,1710; ^1^H-NMR (300 MHz, CDCl_3_) δ: 7.90–8.05 (m, 12H, aromatic and C*H*= protons), 6.75–6.88 (m, 12H, aromatic and C*H*= protons), 4.30–4.38 (m, 4H, aliphatic protons), 2.99–3 (br, 12H, 4 × CH_3_), 2.32–2.36 (m, 4H, aliphatic protons), 1.75–1.80 (m, 4H, aliphatic protons), 1.43–1.45 (m, 4H, aliphatic protons); ^13^C-NMR (75 MHz, CDCl_3_) δ: 196.80 (C=O), 162.95 (−C–O), 131.52, 129.34, 128.70, 127.35, 67.83, 42.67, 29.74, 28.80, 23.62 (−CH_2_−); elemental analysis: expected % (C_46_H_52_N_2_O_4_): C 79.28, H 7.52, N 4.02; found % (C_46_H_52_N_2_O_4_): C 79.02, H 7.47, N 3.98.

### 3.3. Physicochemical Studies

#### 3.3.1. Molecular Properties Prediction-Lipinski “Rule of Five”

Compounds were subjected to molecular properties prediction, drug-likeness by molinspiration [44] (Table 1).

#### 3.3.2. Determination of R_M_ Values

Reversed phase TLC (RP-TLC) was performed on silica gel plates impregnated with 5% (*v*/*v*) liquid paraffin in light petroleum ether. The mobile phase was a methanol/water mixture (70/30, *v*/*v*) [29]. The plates were developed in closed chromatography tanks saturated with the mobile phase at 24 °C. Spots were detected under UV light. Five individual measurements of R_f_ values were recorded and used for the determination of R_M_ using the equation R_M_ = log [(1/R_f_) − 1]. (Table 3).

### 3.4. Biological In Vitro Assays

Each in vitro experiment was performed at least in triplicate and the standard deviation of absorbance was less than 10% of the mean. For the in vitro assays, a stock solution (1% DMSO in the appropriate buffer with the tested compound diluted under sonication) was prepared from which several dilutions were made with the appropriate buffer.

#### 3.4.1. Determination of the Reducing Activity Using the Stable Radical 1,1-Diphenyl-Picrylhydrazyl (DPPH)

To a solution of DPPH (final concentration 0.05 mM) in absolute ethanol an equal volume of the compounds solution in DMSO (stock solution 10 mM) dissolved in ethanol was added. Absolute ethanol was used as a control. The concentrations of the solutions of the compounds were 0.05 and 0.1 mM. The absorbance was recorded at 517 nm at room temperature after 20 and 60 min [29] (Table 2).

#### 3.4.2. Inhibition of Linoleic Acid Lipid Peroxidation

In an aqueous solution spectrophotometrically at 234 nm the production of conjugated diene hydro peroxide by oxidation of sodium linoleate was monitored and recorded according to our previous published experimental conditions [29], using 10 µL of the 16 mM sodium linoleate solution added to the UV cuvette containing 930 μL of 0.05 M phosphate buffer, pH 7.4 pre-thermostated at 37 °C, under air by the addition of 50 μL of 40 mM AAPH solution, which was used as a free radical initiator. Ten microliters (10 μL) of the appropriate solutions of the tested compounds were added in the mixture, whereas same level of DMSO was used as a blank for the measurement of lipid oxidation. The rate of oxidation at 37 °C was monitored by recording the increase in absorption at 234 nm caused by conjugated diene hydro peroxides. The results were compared to the appropriate standard inhibitor Trolox (93%) (Table 2).

#### 3.4.3. In Vitro Lipid Peroxidation Assay

Egg phosphatidylcholine (20 mg) in chloroform (2 mL) was dried under vacuum in a rotary evaporator to give a thin homogeneous film and further dispersed in normal saline (5 mL) with a vortex mixer. The mixture was sonicated to obtain a homogeneous suspension of liposomes. Lipid peroxidation was initiated by adding 0.05 mM ascorbic acid to a mixture containing liposome (0.1 mL), 150 mM potassium chloride, 0.2 mM ferric chloride, and the tested compounds in a total volume of 0.4 mL final concentration of 100 µM. The reaction mixture was incubated for 40 min at 37 °C. After incubation, the reaction was terminated by adding 1 mL of ice-cold 0.25 M hydrochloric acid containing 20% *w*/*v* trichloroacetic acid, 0.4% *w*/*v* of thiobarbituric acid, and 0.05% *w*/*v* butylated hydroxytoluene. After heating at 80 °C for 20 min, the samples were cooled and the pink chromogen was extracted with a constant amount of butan-1-ol, and the absorbance of the upper organic layer was measured at 532 nm [75] (Table 2).

#### 3.4.4. ABTS^•+^—Decolorization Assay for Antioxidant Activity

Stock solutions of the tested compounds in DMSO (10 mM) were diluted. For the study, the ABTS^•+^ solution was diluted with ethanol to an absorbance of 0.70 at 734 nm. Ten milliliter (10 mL) aliquots of each sample were added into the ABTS·^+^ solution and the absorbance was recorded at room temperature [76]. Trolox was used as a standard.

#### 3.4.5. Soybean Lipoxygenase Inhibition Study In Vitro

In vitro study was evaluated as reported previously [29]. The tested compounds as stock solutions were dissolved in DMSO. Ten microliters (10 μL) were incubated at room temperature with sodium linoleate (100 mM) and 0.2 mL of enzyme solution (1/9 × 10^−4^ *w*/*v* in saline) in buffer pH 9 (tris) at room temperature (final volume 1 mL). The conversion of sodium linoleate to 13-hydroperoxylinoleic acid at 234 nm was recorded. The results were compared with the appropriate standard inhibitor NDGA (IC_50_ = 0.5 μM). Several concentrations were used for the determination of IC_50_ values. The results are given in Table 3 expressed as IC_50_ values or % inhibition at 100 μM.

#### 3.4.6. Human h-15-LOX-1 Screening UV Assay [77]

The screening UV assay of h-15-LOX-1 was also studied by the formation of 13(S)-HPODE at 234 nm. The assay buffer consists of HEPES titrated to pH 7.5 using a concentrated aqueous solution of NaOH. Linoleic acid (LA) was used as a substrate. The linear absorbance increase in absence of the inhibitor was set as 100%, whereas the absorbance increase in absence of the enzyme was set to 0%. All experiments were performed in triplicate and the average triplicate values and their standard deviations are plotted. The results for representative compounds are given in Figure 6. The vertical axis describes the residual enzyme activity percent % of the human h-15-LOX-1, whereas the horizontal axis describes the concentration of 50 µM of the compounds. The half maximal inhibitor concentration (IC_50_) of the inhibitors for h-15-LOX-1 was determined using the same assay (Figure 6 and Figure 7).

#### 3.4.7. Inhibition of Acetyl-Cholinesterase

A modified procedure which was followed in our previous publication was performed [29]. A stock solution (10 mM) of the compounds diluted in DMSO was used (final concentration 100 µM). The inhibitory activity was measured by the change in absorbance at 405 nm in phosphate buffer pH 8 (0.1 M. The thiol ester acetylthioline was used as a substrate (0.01 M). It was hydrolyzed by AChE (3.5 U/mL) to produce thiocholine and acetate. The thiocholine reduces DTNB (0.01 M in phosphate 0.1 M pH 7) liberating nitrobenzoate, which absorbs at 405 nm. The absorbance is measured with Perkin-Elmer multilabel plate reader Victor X3. Tacrine was used as the reference compound.

#### 3.4.8. Evaluation of the Cytotoxicity

L929 mouse fibroblasts cells were cultured in EMEM supplemented with 10% horse serum, 2 mM L-glutamine, 1 mM sodium pyruvate, 0.1 mM nonessential amino acids, 1.5 g/L sodium bicarbonate, and 100 μg/mL penicillin–streptomycin at 37 °C in a humidified atmosphere with 5% CO_2_. L929 cells were plated into 24-well plates at a density 5104 cells/well and allowed to attach and grow for 24 h. The supernatant in each well then replaced with medium containing various concentrations (1, 10, 20, 50 and 100 μM) of compounds **a5**–**12**, **c5**–**12**, **di**, and **dii**–**dvi**, which presented the more interesting results in the in vitro experiments [29]. After 24-h incubation, the supernatant was removed and the cells were washed with PBS. The cells were detached with 0.25% trypsin, transferred to FACS tubes, and then centrifuged (1600 rpm for 5 min) and the pellet washed with PBS. After washing, the cells in the pellet were incubated with 5 μL propidium iodide (PI) solution (1 mg/mL) for 1 min. The PI fluorescence (cell death) was determined with flow cytometry, FACS Calibur, Coulter Epics XL-MCL (Beckman, Inc., Mount Holly, NJ, USA). The analysis of flow cytometry data was performed with the WinMDI analysis program (Table 4).

#### 3.4.9. Circular Dichroism Studies

Preparation of Aβ stocks and solutions. Amyloid peptide Aβ40 was purchased from AnaSpec Co. (Fremont, CA, USA) (>95% pure). Aβ40 was gently dissolved without vortexing in Type 1 (Milli-Q) water to a final concentration of 100 μM. Solutions of Aβ in phosphate buffer (PB, 10 mM, pH 7.33) were prepared by adding equal volumes of PB to aliquots of the aqueous solution stock to achieve final concentration of 50 µM. The proper amount of the compounds c**2**, **c3**, **c4**, **di**, **dii**, and **dvi** were added to the Aβ solutions to achieve concentration of 50 μM. The secondary structural changes of Aβ were monitored for 40 days by CD. During this period, the amyloid peptide solutions were kept at an incubator at 33 °C under quiescent conditions [66].

*Circular dichroism measurements***.** CD spectra were recorded on a JASCO J-715 spectropolarimeter (Jasco Co., Tokyo, Japan), at 33 °C in the range of 190–260 nm with a 1 mm path length quartz cuvette. Each spectrum was the average of three scans at a speed of 100 nm·min^−1^ and a resolution of 0.5 nm. Three independent experiments were run for each condition and in each case solutions of plain Aβ were run as control (Figure 8). The analysis of the CD data was performed using the OriginPro 9 program [66].

## 4. Conclusions

In conclusion, **a**, **b**, **c**, and **e** chalcones led to a new and promising class of antioxidant and anti-lipoxygenase enones with anti-AChE activities. In Table 5 are summarized the most significant chalcones in terms of activities. It should to be noticed that the simple chalcone **b4** presents significant inhibitory activity against the 15-human LOX with an IC_50_ value of 9.5 µM, interesting anti-AChE activity and anti-lipid peroxidation behavior. *Bis*-etherified chalcone **c12** is the most potent inhibitor of AChE within the *bis*-etherified *bis*-chalcones following, by **c11** which also is found to present interesting inhibition on 15-human LOX. *Bis*-chalcones **c11** and **c12** were found to combine anti-LOX, anti-AChE, and anti-lipid peroxidation activities. Thus, they can be used as lead multifunctional molecules.

It seems that the anti-lipid peroxidation activity supports the anti-LOX activity for the significantly active *bis*-chalcones. The presence of a double enone group supports better biological results.

Our CD study identified also that *bis*-etherified chalcones **c2** and **c4** are capable of interfering with the aggregation process of Aβ and offer to the AD treatment as anti-AChE and anti-LOX inhibitors.

Since the molecular weight of 29 derivatives was found to be less than 500, these molecules are anticipated to be easily transported, diffused, and absorbed and they are predicted to be orally active, as they obeyed Lipinski’s rule of five. TPSA of the derivatives was observed to be well below the limit of 160 Å and indicated good oral bioavailability. The prediction indicates that all structures with the exception of **c11** (144 Å) can cross BBB and act in CNS. The pharmacokinetic prediction study seems quite interesting and useful to the design and optimization of the pharmacokinetic profile of the new derivatives. As we have recently reported [29], the most probable sites of metabolism for the enzymes CYP1A1 and CYP1A2 are the hydrogens of the **−OCH_2_CH_2_CH_2_O−** linkage. Therefore, it appears possible that the molecules of the **c/e** groups act as prodrugs. Further investigation is in progress to study this possibility.

Mechanistic studies attempting to determine the actual mode of the antioxidative, anti-LOX, and anti-AChE action are under way.

## Figures and Tables

**Table 1:** Molecular properties prediction-Lipinski “Rule of Five”.

Compounds	mi Log P ^a^	TPSA ^b^	n Atoms	n ^c^ O, N	n ^d^ OH, NH	n Violations	Nrotb ^e^	mol. Wt ^f^	Volume ^g^	^h^ LogBB
a1 *	5.79	46.53	26	3	1	1	6	409.28	324.95	0.55275
a2 *	5.06	46.53	24	3	1	1	5	316.36	290.26	0.483
a3 *	3.95	40.54	22	3	1	0	5	293.37	283.19	0.3135
a4 *	4.31	37.3	21	2	1	0	3	274.32	253.86	0.43115
**a5**	3.05	37.3	16	2	1	0	3	230.04	200.58	0.1940
**a6**	2.43	49.33	20	3	2	0	3	265.31	245.06	−0.03325
**a7**	3.85	37.3	19	2	1	0	4	250.3	237.29	0.31955
**a8**	4.4	37.3	20	2	1	0	4	264.32	253.85	0.38155
**a9**	4.32	37.3	20	2	1	0	4	329.19	255.17	0.3955
**a10**	2.41	50.44	16	3	1	0	3	214.22	191.44	−0.01035
**a11**	3.81	83.12	22	5	1	0	5	295.29	260.62	−0.17895
**a12**	3.32	33.37	15	2	1	0	2	200.24	189.02	0.23795
**a13**	3.33	37.3	17	2	1	0	3	224.26	209.87	0.2498
**b1**	6.21	46.53	26	3	1	1	6	409.28	324.95	0.62405
**b2**	5.48	46.53	24	3	1	1	5	316.36	290.26	0.5543
**b3**	4.37	40.54	22	3	1	0	5	293.37	283.19	0.3848
**b4**	4.73	37.3	21	2	1	0	3	274.32	253.86	0.50245
**b7**	4.27	37.3	19	2	1	0	4	250.3	237.29	0.39085
**b8**	4.82	37.3	20	2	1	0	4	264.32	253.85	0.45285
**b9**	4.74	37.3	20	2	1	0	4	239.19	255.17	0.4668
**b11**	4.23	83.12	22	5	1	0	5	295.29	260.62	−0.10765
**b13**	3.75	37.3	17	2	1	0	3	224.26	209.87	0.3211
**di**	3.47	52.61	22	4	0	0	7	298.34	278.11	0.15095
Dii *	3.74	52.61	23	4	0	0	8	312.37	294.92	0.2083
**diii**	4.01	52.61	24	4	0	0	9	326.39	311.72	0.25325
**div**	4.52	52.61	25	4	0	0	10	340.42	328.52	0.33385
**dv**	5.03	52.61	26	4	0	1	11	354.45	345.32	0.416
**dvi**	5.53	52.61	27	4	0	1	12	368.74	362.12	0.50435
**dvii**	6.04	52.61	28	4	0	1	13	382.5	378.93	0.5803
c1 *	9.72	71.08	55	6	0	2	18	858.62	689.61	1.2791
c2 *	9.42	71.08	51	6	0	2	16	672.78	620.23	1.1396
c3 *	8.66	59.09	47	6	0	2	16	626.8	606.09	0.8007
c4 *	8.98	52.61	45	4	0	2	12	588.7	547.43	1.03755
**c5**	7.13	52.61	35	4	0	2	12	500.64	440.87	0.5648
**c6**	5.82	76.66	43	6	2	2	12	570.69	529.83	0.2561
**c7**	8.55	52.61	41	4	0	2	14	540.66	514.28	0.81435
**c8**	9.04	52.61	43	4	0	2	14	568.71	547.4	0.93835
**c9**	8.98	52.61	43	4	0	2	14	698.45	550.05	0.9678
**c10**	5.85	78.89	35	6	0	1	12	468.5	422.58	0.1563
**c11**	8.5	144.26	47	10	0	2	16	630.65	560.95	-0.1812
**c12**	6.29	78.89	37	6	0	1	12	496.56	455.7	0.3113
**c13**	7.69	52.61	37	4	0	1	12	488.58	459.44	0.67485
**ei**	8.51	59.09	46	6	0	2	15	612.77	589.29	0.74335
**eiii**	8.79	59.09	48	6	0	2	17	640.82	622.89	0.93565
**eiv**	9	59.09	49	6	0	2	18	654.85	639.69	1.0178
**ev**	9.17	59.09	50	6	0	2	19	668.88	656.5	1.00995
**evi**	9.31	59.09	51	6	0	2	20	682.9	673.3	1.0921
**evii**	9.44	59.09	52	6	0	2	21	696.93	690.1	1.17425
**tacrine**	3.05	38.91	15	2	2	0	0	198.27	191.53	1.053785
**NDGA**	3.48	80.91	22	4	4	0	5	302.37	287.90	1.6613

^a^ Logarithm of partition coefficient between *n*-octanol and water (milog P); ^b^ Topological polar surface area (TPSA); ^c^ Number of hydrogen bond acceptors (n-ON); ^d^ Number of hydrogen bond donors (n-OHNH); ^e^ Number of rotatable bonds (n-rotb); ^f^ Molecular weight; ^g^ Molecular volume; ^h^ blood brain barrier; * these compounds referred in [29].

**Table 2:** Antioxidant activity of the tested compounds % reducing activity (RA %); ABTS^•+^—decolorization assay%; % anti-lipid peroxidation (AAPH%); and % inhibition of lipid peroxidation of liposomes (% Inhb. of liposomes LP).

Compound	RA% 100 μM 20 min	RA% 100 μM 60 min	ABTS^•+^ Inhb. % @ 100 μM	AAPH% @ 100 μM	% Inhb. of Liposomes LP @ 100 μM
a1 *	1	6	No	*	nt
a2 *	3	4	No	*	nt
a3 *	18	17	12	*	nt
a4 *	7	3	No	*	nt
**a5**	17	3	No	23	nt
**a6**	15	2	No	45	nt
**a7**	19	6	12	31	nt
**a8**	6	2	No	32	nt
**a9**	10	1	No	33	nt
**a10**	23	10	2	24	nt
**a11**	7	3	14	55	nt
**a12**	24	9	6	45	nt
**a13**	7	3	No	38	nt
**b1**	46	0	40	89	nt
**b2**	45	15	No	44	nt
**b3**	72	15	96	100	nt
**b4**	30	20	No	82	nt
**b7**	13	0	5	29	nt
**b8**	17	3	12	25	nt
**b9**	20	8	No	17	nt
**b11**	4	2	No	31	nt
**b13**	10	0	No	15	nt
**di**			No	22	nt
Dii *	No	no	No	20	nt
**diii**			No	29	nt
**div**			No	30	nt
**dv**			No	18	nt
**dvi**			No	12	nt
**dvii**			No	27	nt
c1 *	36	55	no	42	nt
c2 *	No	8	no	22	2
c3 *	17	14	99	70	81
c4 *	19	50	no	95	3
**c5**	No	no	No	46	nt
**c6**	No	No	No	100	nt
**c7**	No	no	No	82	nt
**c8**	No	no	No	75	nt
**c9**	No	no	No	32	nt
**c10**	No	no	11	77	nt
**c11**	No	no	No	68	nt
**c12**	No	no	17	68	74
**c13**	No	no	No	71	nt
**ei**	No	no	98	71	4
**eiii**	No	no	No	47	nt
**eiv**	No	no	73	72	77
**ev**	No	no	25	14	nt
**evi**	No	no	79	64	64
**evii**	No	no	88	6	nt
**Trolox**	Nt	nt	96	93	69
**Tacrine**	Nt	nt	88	nt	nt
**NDGA**	81	83	nt	nt	nt

No, no activity under the reported experimental conditions. Means within each column differ significantly (*p* < 0.05); nt, not tested; * [29].

**Table 3:** In vitro inhibition of soybean lipoxygenase (IC_50_ μM or LOX Inh. %) and in vitro inhibition of acetyl-cholinesterase (IC_50_ μM or AChE Inh. %); experimentally-determined lipophilicity **R_M_**; theoretically calculated lipophilicity values cLogP.

Compound	% LOX Inhb. @ 100 μM/IC_50_ μM	% AChE Inhb. @ 100 μM/IC_50_ μM	R_M_ ± (SD) ^¥^	cLogP
a1 *	14	7	0.34	5.51
a2 *	10	26	0.36	5.06
a3 *	56/100 μM	50/100 μM	−0.69	3.58
a4 *	44	25	−0.26	4.13
**a5**	9	38	−0.62	2.60
**a6**	60	Νο	−0.65	1.91
**a7**	4	63/87 μM	−0.64	3.41
**a8**	14	57/100 μM	−0.68	3.81
**a9**	16	48	−0.82	3.90
**a10**	23	27	−0.06	2.13
**a11**	36	56/100 μM	−1.09	3.15
**a12**	2	42	−0.81	2.63
**a13**	23	25	−0.73	2.96
**b1**	89/56 μM	89/100 μM	−0.91	5.97
**b2**	44	44	0.04	5.52
**b3**	100/57 μM	100 ^#^	−0.66	4.04
**b4**	82/65 μM	82 ^#^	−0.03	4.59
**b7**	29	29	−0.85	3.87
**b8**	25	25	−0.79	4.27
**b9**	16	16	−0.37	4.36
**b11**	31	31	−0.91	3.61
**b13**	14	14	0.43	3.42
**di**	12	22	0.67	3.31
Dii *	no	20	−0.23	3.68
**diii**	41	29	−0.37	3.97
**div**	17	29	0.23	4.49
**dv**	7	18	−0.53	5.02
**dvi**	17	12	0.32	5.59
**dvii**	5	27	−0.36	6.08
c1 *	41	23	−0.68	10.88
c2 *	100/55 μM	71/49 μM	−0.89	7.92
c3 *	93/56 μM	95/52 μM	0.77	9.03
c4 *	100/55 μM	58/100 μM	0.43	5.98
**c5**	32	51/100 μM	0.43	5.98
**c6**	98/54 μM	Νο	−0.97	5.54
**c7**	41	94/56 μM	−0.77	7.59
**c8**	26.5	100/58 μM	−0.85	8.39
**c9**	24.5	85/74 μM	−0.86	8.58
**c10**	57/85 μM	69/76 μM	0.7	5.04
**c11**	95/50 μM	100/52 μM	−0.88	7.08
**c12**	52/96 μM	100/48 μM	−0.96	6.04
**c13**	76/65 μM	50.5/100 μM	0.34	6.69
**ei**	77/62.5 μM	71/57.5 μM	0.56	7.55
**eiii**	27	47	−0.07	8.21
**eiv**	23	72/76 μM	0.13	8.74
**ev**	7	14	Nd	9.27
**evi**	65/76 μM	64/62 μM	Nd	9.79
**evii**	12	6/85 μM	Nd	10.4
**Tacrine**		98/0.03 μM		
**NDGA**	93/0.5 μM			

* [29] ^#^ % activity at 0.001 µM; ^¥^ SD < 10%; Means within each column differ significantly (*p* < 0.05); nd: not determined under the experimental conditions.

**Table 4:** Cytotoxicity of chalcones and *bis*-etherified chalcones on L929 cells (24-h incubation) expressed as PI % values.

Compound	1 μM Average (% pi) ± σ	10 μM Average (% pi) ± σ	20 μM Average (% pi) ± σ	50 μM Average (% pi) ± σ	100 μM Average (% pi) ± σ
a1 *	1 ± 0.6	5 ± 2.8	5.5 ± 0.71	32 ± 4.9	32 ± 4.9
a2 *	1 ± 0.5	3.5 ± 0.7	27.5 ± 8.9	63 ± 11.2	63 ± 11.2
a3 *	14 ± 3.5	15 ± 0.8	19 ± 1.4	14 ± 0.7	14 ± 0.7
a4 *	8.5 ± 2.1	12 ± 4.2	29.5 ± 0.6	51 ± 9.9	51 ± 9.9
**a5**	11.36 ± 0.62	Νο ± No	28.32 ± 2.91	36.15 ± 2.33	36.15 ± 2.33
**a6**	1.71 ± 2.08	1.66 ± 0.59	3.25 ± 2.6	6.67 ± 1.35	6.67 ± 1.35
**a7**	6.85 ± 3.12	9.27 ± 1.9	9.24 ± 6.49	60.95 ± 0.01	60.95 ± 0.01
**a8**	1.39 ± 0.1	2.95 ± 1.23	1.84 ± 2.05	8.15 ± 0.34	8.15 ± 0.34
**a9**	0.55 ± 0.09	0.59 ± 0.83	2.7 ± 0.68	7.59 ± 0.13	7.59 ± 0.13
**a10**	3.03 ± 0.88	2.25 ± 0.38	2.39 ± 1.08	17.15 ± 0.32	17.15 ± 0.32
**a11**	3.13 ± σ 2.43	6.26 ± 3	18.81 ± 2.57	67.84 ± 6.45	67.84 ± 6.45
**a12**	0.88 ± 0.31	3.25± 2.11	8.12 ± 0.32	63.54 ± 6.16	63.54 ± 6.16
**di**	4.94 ± 1.02	9.05 ± 0.58	16.78 ± 5.61	33.22 ± 9.65	33.22 ± 9.65
dii *	9 ± 4.5	8.5 ± 4.24	13 ± 4.5	42 ± 3.5	42 ± 3.5
**diii**	14.06 ± 1.36	20 ± 3.12	20.76 ± 3.51	32.14 ± 6.99	32.14 ± 6.99
**div**	4.89 ± 0.51	8.56 ± 3.97	11.86 ± 2.98	17.05 ± 1.46	17.05 ±1.46
**dv**	4.47 ± 0.59	6.83 ± 2.46	8.05 ± 3.58	9.69 ± 0.57	9.69 ± 0.57
**dvi**	0.37 ± 0.52	1.92 ± 1.02	1.79 ± 1.53	2.55 ± 0.5	2.55 ± 0.5
c1 *	38.5 ± 6.9	49.5 ± 7.8	53.5 ± 0.71	74 ± 5.6	74 ± 5.6
c2 *	17 ± 1.4	29 ± 4.2	43 ± 2.8	55 ± 4.2	55 ± 4.2
c3 *	81 ± 0.8	90 ± 1	100 ± 0	100 ± 0	100 ± 0
c4 *	29.5 ± 3.5	42 ± 4.6	46 ± 5.6	67 ± 9.2	67 ± 9.2
**c5**	0.32 ± 0.45	0.97 ± 0.2	3.02 ± 1.36	7.26 ± 0.3	7.26 ± 0.3
**c6**	1.12 ± 1.15	2.2 ± 1.66	1.16 ± 0.81	3.03 ± 0.02	3.03 ± 0.02
**c7**	22.5 ± 11.64	28.73 ± 11.78	25.4 ± 8.42	28.84 ± 5.62	28.84 ± 5.62
**c8**	35.72 ± 10.23	29.05 ± 9.62	35.69 ± 5.85	38.7 ± 4.96	38.7 ± 4.96
**c9**	12.5 ± 10.31	15.88 ± 6.31	28.76 ± 2.74	29.53 ± 6.87	29.53 ± 6.87
**c10**	18.10 ± 11.82	13.48 ± 10.36	25.93 ± 9.73	32.11 ± 9.38	32.11 ± 9.38
**c11**	6.4 ± 0.4	19.07 ± 7.23	34.63 ± 0.31	64.53 ± 3.07	64.53 ± 3.07
**c12**	13.22 ± 0.64	31.31 ± 4.84	53.79 ± 16.95	84.03 ± 2.25	84.03 ± 2.25

* ref [29]; Means within each column differ significantly (*p* < 0.05).

**Table 5:** Summary comparison table of biological activities.

Compound	% Soybean LOX Inhb. @ 100 μM/IC_50_ μM	% AChE Inhb. @ 100 μM/IC_50_ μM	h-15-LOX-1 Inhb. IC_50_ μM	AAPH% @ 100 μM	% Inhb. of Liposomes LP @ 100 μM
**b3**	100/57 μM	100 ^#^			
**b4**	82/65 μM	82 ^#^	9.5 μM	82	
c2 *	100/55 μM	71/49 μM			
c3 *	93/56 μM	95/52 μM		70	81
c4 *	100/55 μM	58/100 μM		95	
**c11**	95/50 μM	100/52 μM	(2nd potent at 50 μM)	68	
**c12**	52/96 μM	100/48 μM		68	74

* ref [29]; ^#^ % activity at 0.001 µM.

## Abbreviations

AA	Arachidonic Acid
AAPH	2,2′-Azo*bis*-(2-amidinopropane) hydrochloride
DPPH	2,2-Diphenyl-1-picrylhydrazyl radical
LOX	Lipoxygenase
NDGA	Nordihydroguaiaretic acid
RPTLC	Reverse-phase thin layer chromatography
TCA	Trichloroacetic acid
